# Double Helix of Icosahedra Structure and Spin Glass Magnetism of the δ-Co_2.5_Zn_17.5–*x*_Mn_*x*_ (*x* = 0.4–3.5) Pseudo-Binary Alloys

**DOI:** 10.1021/acs.inorgchem.4c00686

**Published:** 2024-05-20

**Authors:** Amit Mondal, Riju Dey, Andreja Jelen, Primož Koželj, Sandip Kumar Kuila, Rahul Pan, Stanislav Vrtnik, Jože Luzar, Magdalena Wencka, Julia Petrović, Peter Mihor, Zvonko Jagličić, Anton Meden, Partha Pratim Jana, Janez Dolinšek

**Affiliations:** † Department of Chemistry, Indian Institute of Technology, Kharagpur 721302, India; ‡ J. Stefan Institute, Jamova 39, SI-1000 Ljubljana, Slovenia; § Faculty of Mathematics and Physics, University of Ljubljana, Jadranska 19, SI-1000 Ljubljana, Slovenia; ∥ Institute of Molecular Physics, Polish Academy of Sciences, Smoluchowskiego 17, PL-60-179 Poznań, Poland; ⊥ Faculty of Civil and Geodetic Engineering, Institute of Mathematics, Physics and Mechanics & University of Ljubljana, Jadranska 19, SI-1000 Ljubljana, Slovenia; # Faculty of Chemistry and Chemical Technology, University of Ljubljana, Večna pot 113, SI-1000 Ljubljana, Slovenia

## Abstract

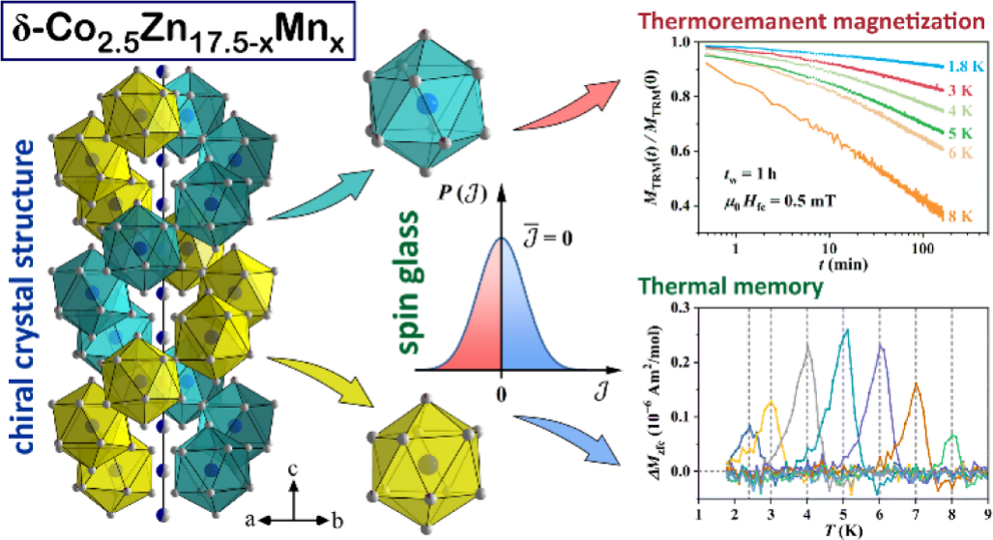

We have synthesized δ-Co_2.5_Zn_17.5–*x*_Mn_*x*_ (*x* = 0.4–3.5) pseudo-binary alloys of 10 different compositions by a high-temperature solid-state synthetic route, determined their crystal structures and the Mn substitution pattern, and estimated the existence range of the δ-phase. The alloys crystallize in two chiral enantiomorphic space groups *P*6_2_ and *P*6_4_, where the basic atomic polyhedron of the chiral structure is an icosahedron and the neighboring icosahedra share vertices to form an infinitely long double helix along the hexagonal axis (like in the δ-Co_2.5_Zn_17.5_ parent binary phase). The alloys are pure δ-phase up to the Mn content *x* ≈ 3.5. The Mn atoms partially substitute Zn atoms at particular crystallographic sites located on the icosahedra. The study of magnetism was performed on the Co_2.5_Zn_17.1_Mn_0.4_ alloy with the lowest Mn content. Contrary to the expectation that structural chirality may induce the formation of a nontrivial magnetic state, a spin glass state with no relation to the structural chirality was found. The magnetic sublattice contains all of the necessary ingredients (randomness and frustration) for the formation of a spin glass state. Typical out-of-equilibrium dynamic phenomena of a spin system with broken ergodicity were detected below the spin freezing temperature *T*_f_ ≈ 8 K.

## Abstract 2

In a search for structural chirality-induced nontrivial magnetic states, we have synthesized the δ-Co_2.5_Zn_17.5−*x*_Mn_*x*_ (*x* = 0.4−3.5) pseudo-binary chiral alloys and determined their crystal structures, Mn substitution pattern, and magnetic properties. A spin glass state with typical broken ergodicity phenomena was found, being in no relation to the structural chirality.

## 1 Introduction

Chiral crystal structures are quite rare in intermetallic compounds. Chirality refers to the situation where an object and its mirror image cannot overlap each other by spatial rotation and translation. Chirality is related to particular symmetries, which exclude a mirror plane, a center of inversion, and any rotation-reflection axes so that only proper symmetry elements (translations, rotations, and compositions of these) are allowed.^1,2^ In terms of crystallographic space groups, an object is chiral when it has no orientation-reversing symmetries so that its proper symmetry group is equal to its full symmetry group.^3^ Out of the overall 230 space groups, the subgroup of 65 space groups (denoted as Sohncke groups) describes chiral crystal structures. Known examples of chiral intermetallic crystals are the Mg_2_Ni-type structures that crystallize in the space group *P*6_2_2_2_,^4^ Ir_3_Zr_5_ in *P*6_1_2_2_,^5^ RuZn_6_ in *P*4_1_3_2_,^6^ δ-Co_2.5_Zn_17.5_ (equivalent to δ-Co_2_Zn_15_) in *P*6_2_,^7^ and ternary compounds Co_*x*_Zn_*y*_Mn_*z*_ (*x* + *y* + *z* = 20, with a representative Co_8_Zn_9_Mn_3_) that crystallize in the cubic β-Mn type structure with two chiral enantiomers *P*4_1_3_2_ and *P*4_3_3_2_.^8^ Structural chirality is considered to play an important role in the electronic and magnetic properties of these systems, as it affects the band structure and thus the electronic density of states, and is involved in the formation of spirally ordered magnetic states such as helimagnetic, conical, skyrmion, and chiral spin soliton lattices.^9−11^ Examples are the Co_*x*_Zn_*y*_Mn_*z*_ (*x* + *y* + *z* = 20) compounds that were reported to exhibit magnetic skyrmion spin textures.^8,12,13^

In a search for nontrivial magnetic states in intermetallic compounds with a chiral crystal structure, we have investigated the pseudo-binary δ-phase system Co_2.5_Zn_17.5–*x*_Mn_*x*_ (*x* = 0.4–3.5), derived from the δ-Co_2.5_Zn_17.5_ parent binary phase, which is the first example of a chiral structure that crystallizes in the acentric space group *P*6_2_.^7^ According to the Co–Zn phase diagram,^14^ the δ-Co_2.5_Zn_17.5_ phase is located in the Zn-rich part, approximately at the Zn concentration between 86 and 89 atom %, and is stable below 690 °C. Its unit cell is hexagonal, containing 60 atoms, of which 7.3 are Co (the compound is not stoichiometric). The basic atomic polyhedron of the structure is a highly regular icosahedron made of 12 Zn atoms with a Co atom in the icosahedron center. The neighboring icosahedra share vertices to form an infinitely long double helix along the hexagonal axis.^7^ The δ-Co_2.5_Zn_17.5_ structure is depicted in Figure S1a in the Supporting Information. In the investigation of the pseudo-binary δ-Co_2.5_Zn_17.5–*x*_Mn_*x*_ system, we have studied the substitution pattern of Mn in the parent δ-Co_2.5_Zn_17.5_ phase by determining the crystal structure and chemical composition for a set of 10 Mn concentrations ranging from 2 to 17 atom %. The results reveal that the crystal symmetry remains chiral *P*6_2_ or its enantiomer *P*6_4_ in the entire substitution range and the Mn atoms partially substitute Zn atoms at particular crystallographic sites located on the icosahedra. A detailed magnetic study was then conducted for the lowest Mn-containing composition *x* = 0.4.

## 2 Materials Synthesis and Structural Characterization

### 2.1 Synthesis

The δ-Co_2.5_Zn_17.5–*x*_Mn_*x*_ (*x* = 0.4–3.5) crystals of 10 different compositions (denoted as c1 to c10, where the relation between the composition denotation and the Mn content *x* is given in Table 1) were synthesized by high-temperature solid-state synthetic route starting from pure elements Zn (99.999%), Co (99.98%), and Mn (99.98%), all from Alfa Aesar. Here, the low-melting Zn (melting point 693 K) serves as a self-flux for the high-melting elements Co (1768 K) and Mn (1519 K). Samples (0.3 g each) of precisely weighted metals were loaded into one-end sealed silica tubes (0.8 cm diameter and 12 cm length) and then purged with argon gas. After evacuation, the other end of the tube was sealed under a high vacuum of 10^–5^ mbar. The sealed tubes were placed in a programmable temperature furnace. The temperature was first raised up to 1223 K at the rate of 1 K/min and kept at that temperature for 600 min. Subsequently, the furnace was slowly cooled down to 723 K at the rate of 0.2 K/min and the reaction mixtures were annealed at that temperature for ∼4.5 days. Finally, the furnace was slowly cooled to 473 K at the rate of 0.08 K/min to grow single crystals and then turned off, allowing the furnace to come to room temperature (RT). Silvery ingots with metallic luster were obtained from each load. They were crushed using an agate mortar and pestle. The resulting materials were stable at ambient conditions and suitable for single-crystal measurements.

**Table 1:** Loaded Compositions of the δ-Co_2.5_Zn_17.5–*x*_Mn_*x*_ (*x* = 0.4–3.5) Samples

composition code	Mn content *x*	loaded composition	loaded composition (atom %)
c1	0.4	Co_2.5_Zn_17.1_Mn_0.4_	Co_12.5_Zn_85.5_Mn_2.0_
c2	0.6	Co_2.5_Zn_16.9_Mn_0.6_	Co_12.5_Zn_84.5_Mn_3.0_
c3	1.0	Co_2.5_Zn_16.5_Mn_1.0_	Co_12.5_Zn_82.5_Mn_5.0_
c4	1.1	Co_2.5_Zn_16.4_Mn_1.1_	Co_12.5_Zn_82.0_Mn_5.5_
c5	1.3	Co_2.5_Zn_16.2_Mn_1.3_	Co_12.5_Zn_81.0_Mn_6.5_
c6	1.7	Co_2.5_Zn_15.8_Mn_1.7_	Co_12.5_Zn_79.0_Mn_8.5_
c7	1.9	Co_2.5_Zn_15.6_Mn_1.9_	Co_12.5_Zn_78.0_Mn_9.5_
c8	2.1	Co_2.5_Zn_15.4_Mn_2.1_	Co_12.5_Zn_77.0_Mn_10.5_
c9	3.0	Co_2.5_Zn_14.5_Mn_3.0_	Co_12.5_Zn_72.5_Mn_15.0_
c10	3.5	Co_2.5_Zn_14.0_Mn_3.5_	Co_12.5_Zn_70.0_Mn_17.5_

### 2.2 Structure Solution and Refinement by Single-Crystal X-ray Diffraction

Single-crystal X-ray diffraction (SCXRD) using monochromatic Mo Kα radiation was employed to determine the structures. APEX3 software was used to reduce the data. All structure solutions and refinements were done using JANA2006 software package.^15^ The Co_2.5_Zn_17.5–*x*_Mn_*x*_ structures were solved based on the reported binary δ-Co_2.5_Zn_17.5_ structure.^7^ During the structure solution, the *P*6_2_ space group was assigned to the structure, creating 11 independent crystallographic sites (one 3*a* site, one 3*b* site, and nine 6*c* sites) in the unit cell. We assigned the sites as M1:3*b*, M2:6*c*, M3:6*c*, M4:6*c*, M5:6*c*, M6:6*c*, M7:6*c*, M8:3*a*, M9:6*c*, M10:6*c*, and M11:6*c*. Some statistical parameters (*R*(*F*), *R*(*F*^2^), goodness-of-fit (GOF), *w*R**(*F*^2^)) related to the structural refinement were monitored during the entire course of refinement. As the structure adopts a chiral space group, the refinement procedure was also repeated in the enantiomorphic space group *P*6_4_ and Flack parameters for the *P*6_2_ and *P*6_4_ were calculated to find the absolute structure.

In the following, we describe the refinement procedure for the sample of c2 composition (loading composition Co_2.5_Zn_16.9_Mn_0.6_, or Co_12.5_Zn_84.5_Mn_3.0_, in atom %). The initial stage of refinement after producing 11 independent sites has given the *R*(*F*) value of 8.39%. As the phase is Zn-rich, we have first assigned all sites to the heavier Zn atoms, giving *R*(*F*) of 6.86% after refinement. To find the distribution of Co and Mn over the sites initially set populated by Zn, but showing relatively high isotropic atomic displacement parameters (APDs) is generally difficult due to similar X-ray scattering factors of the three elements, so we have adopted the following strategy. We made a comparison to the binary δ-Co_2.5_Zn_17.5_ parent phase,^7^ for which the refinement was made against the neutron diffraction data that unambiguously distinguish Zn and Co. We assumed that Co enters only those sites as in the δ-Co_2.5_Zn_17.5_ parent phase. In this way, all Co from the chemical formula was already used, so it was reasonable to assume that Mn predominantly substitutes Zn at other sites. This is supported by considering that the Pauling electronegativity of Mn(1.55) is closer to that of Zn(1.65) than to Co(1.88). Using this strategy, we have refined the occupancies of all sites independently. The site occupancy factor (SOF) of M11 (6c) site was considerably less than 1 and also the isotropic ADP of ∼0.011 Å^2^ was slightly high. So we have assigned the M11 site as fully occupied by Co and the subsequent refinement has given the *R*(*F*) value of 6.36%. The isotropic ADP of 0.013 Å^2^ of the M8 (3a) site was also slightly high and the SOF was less than 1. We have considered this site as mixed Zn/Co and the subsequent refinement has given *R*(*F*) of 6.32%. The M9 (6c) site showed quite a high isotropic ADP of 0.017 Å^2^, and its SOF was less than 1, so we have refined this site by partially mixing with Mn, keeping the overall SOF to unity, which has reduced the *R*(*F*) to 6.25%. Similarly, all of the remaining sites were refined, of which only the M4 and M6 sites have shown SOF less than unity. These two sites were assumed to be Zn/Mn mixed and the subsequent refinement resulted in the *R*(*F*) value of 6.20%. At the last stage of the refinement, the anisotropic (harmonic) ADPs of all atoms were taken into account and the isotropic extinction correction was applied, after which the *R*(*F*^2^) parameter value has converged to 2.56%. The Flack parameter value for the refinement in *P*6_2_ amounted to −0.09(7). Repeating the refinement in *P*6_4_ has yielded the Flack parameter 1.09(7), supporting that the absolute structure is *P*6_2_. The refinement procedure for the samples of other compositions was performed along the same steps. The X-ray crystallographic data for the crystals of compositions c2 (loading composition Co_2.5_Zn_16.9_Mn_0.6_), c5 (Co_2.5_Zn_16.2_Mn_1.3_), and c9 (Co_2.5_Zn_14.5_Mn_3_) are listed in Table 2. The structural data of all investigated compositions c1 to c10 are also available as CIF files, deposited in the Cambridge Structural Database as the numbers CSD 2330880, 2330885–2330893.

**Table 2:** X-ray Crystallographic Data for the Crystals of Loading Compositions Co_2.5_Zn_16.9_Mn_0.6_ (c2), Co_2.5_Zn_16.2_Mn_1.3_ (c5), and Co_2.5_Zn_14.5_Mn_3_ (c9) (CSD Deposition Numbers 2330887, 2330885, and 2330886, Respectively)

crystallographic data	c2	c5	c9
chemical formula	Co_2.434_Zn_16.934_Mn_0.632_	Co_2.398_ Zn_16.245_Mn_1.357_	Co_2.406_Zn_14.481_Mn_3.113_
chemical formula (atom %)	Co_12.17_Zn_84.67_Mn_3.16_	Co_11.99_Zn_81.22_Mn_6.79_	Co_12.03_Zn_72.41_Mn_15.56_
EDS formula (atom %)	Co_12.0_Zn_84.7_Mn_3.3_	Co_12.6_Zn_79.7_Mn_7.7_	Co_11.8_Zn_73.7_Mn_14.5_
loaded composition (atom %)	Co_12.5_Zn_84.5_Mn_3.0_	Co_12.5_Zn_81.0_Mn_6.5_	Co_12.5_Zn_72.5_Mn_15.0_
Pearson symbol	*hP*60		
crystal system	hexagonal		
space group; *Z*	*P*6_2_(171); 3	*P*6_2_(171); 3	*P*6_4_(172); 3
*a* (Å)	11.2727(6)	11.3260(6)	11.3264(3)
*c* (Å)	7.7155(8)	7.7395(8)	7.6788(5)
*V* (Å^3^)	849.08(11)	859.80(11)	853.12(6)
ρ_calc_ (g cm^–3^)	7.541	7.4045	7.3551
μ (mm^–1^)	39.18	38.00	36.54
crystal size (μm^3^)	140 × 80 × 40	130 × 30 × 30	60 × 40 × 30
crystal color	silvery with metallic luster		
data collection	four-circle diffractometer		
diffractometer	Bruker Photon II		
radiation; wavelength (Å)	Mo Kα; 0.71073		
monochromator	graphite		
*T* (K)	293		
θ_min_ – θ_max_ (deg)	2.09–35.62	2.08–34.90	2.08–35.68
reflns measured	10,498	13,496	7496
index range	–18 ≤ *h* ≤ 16	–18 ≤ *h* ≤ 18	–18 ≤ *h* ≤ 15
	–18 ≤ *k* ≤ 16	–18 ≤ *k* ≤ 18	–15 ≤ *k* ≤ 18
	–12 ≤ *l* ≤ 12	–12 ≤ *l* ≤ 12	–9 ≤ *l* ≤ 12
data reduction/abs. correction	multiscan		
unique reflns	2598	2494	2487
*R*_int_	0.0411	0.054	0.048
structure solution/refinement	JANA2006 package program		
structure solution	superflip		
no. reflns used	3674	4693	3025
no. variables	98	99	101
observed reflns (*I* > 3σ(*I*))	2215	2250	2179
*R*(*F*^2^ > 2σ(*F*^2^))	0.026	0.025	0.041
*R*(*F*) (all data)	0.0356	0.0292	0.0478
*w*R**(*F*^2^) (all data)	0.056	0.061	0.091
GOF (all)	1.08	1.17	1.45
Δρ_min_/Δρ_max_ (eÅ^–3^)	–0.79/0.81	–0.88/0.66	–1.08/1.13
flack parameter	–0.09(7)	0.03(6)	0.07(9)

The final chemical decorations of the 11 independent sites for the c2 composition were the following: M1: Zn1 (3*b*), M2: Zn2 (6*c*), M3: Zn3 (6*c*), M4: Zn4/Mn4 (6*c*), M5: Zn5 (6*c*), M6: Zn6/Mn6 (6*c*), M7: Zn7 (6*c*), M8: Zn8/Co8 (3*a*), M9: Zn9/Mn9 (6*c*), M10: Zn10 (6*c*), and M11: Co11 (6*c*). The Wyckoff sites, atomic coordinates, site occupancies, and isotropic ADPs for the refined c2 composition (Co_12.17_Zn_84.67_Mn_3.16_, in atom %) are given in Table 3, whereas the interatomic distances (<3.0 Å) for each site are given in Table S1 in the Supporting Information.

**Table 3:** Wyckoff Sites, Atomic Coordinates, Site Occupancies, and Equivalent Isotropic Displacement Parameters for the Refined c2 Composition (Co_12.17_Zn_84.67_Mn_3.16_)

site	atom	Wyck.	*x*	*y*	*z*	occupancy	*U*_eq_ (Å^2^)
M1	Zn1	3*b*	0.5	0.5	0.13836(11)	1	0.0145(3)
M2	Zn2	6*c*	0.24302(5)	0.31284(6)	0.13355(8)	1	0.0176(2)
M3	Zn3	6*c*	0.66117(5)	0.38276(5)	0.13393(8)	1	0.01378(17)
M4	Zn4/Mn4	6*c*	0.42371(5)	0.26826(5)	0.31083(7)	0.908(12)/0.092	0.0118(2)
M5	Zn5	6*c*	0.48214(5)	0.11081(6)	0.10951(8)	1	0.0134(2)
M6	Zn6/Mn6	6*c*	0.48296(5)	0.10830(5)	0.50770(7)	0.930(10)/0.070	0.0122(2)
M7	Zn7	6*c*	0.42673(5)	0.31311(5)	0.63886(8)	1	0.01353(19)
M8	Zn8/Co8	3*a*	0	0	0.96610(12)	0.566(16)/0.434	0.0128(3)
M9	Zn9/Mn9	6*c*	0.02787(6)	0.81031(6)	0.14493(9)	0.846(10)/0.154	0.0174(2)
M10	Zn10	6*c*	0.22684(5)	0.06843(6)	0.12092(8)	1	0.01743(19)
M11	Co11	6*c*	0.42471(6)	0.28526(7)	0.97048(12)	1	0.00703(17)

The absolute structures of the 10 investigated compositions c1–c10 were derived from the values of the Flack parameter for the refinements in *P*6_2_ and *P*6_4_, as collected in Table 4. Here we recall that if the Flack parameter value is near 0, with a small standard uncertainty, the absolute structure given by the refinement is likely correct, and if the value is near 1, then the inverted structure is likely correct. If the value is near 0.5, the crystal may be racemic or twinned. Table 4 reveals that the compositions c1, c2, c4, c5, and c8 crystallize in the space group *P*6_2_, while the remaining compositions c3, c6, c7, c9, and c10 adopt the space group *P*6_4_. There is no clear indication of twinning for any of the investigated crystals.

**Table 4:** Flack Parameters of the Compositions c1–c10 for the Refinements in *P*6_2_ and *P*6_4_^a^

composition code	Mn content *x* (loaded)	Flack parameter (*P*6_2_)	Flack parameter (*P*6_4_)
**c1**	0.4	–0.1(3)	1.0(3)
**c2**	0.6	–0.09(7)	1.09(7)
c3	1.0	1.05(7)	–0.05(7)
**c4**	1.1	–0.01(5)	1.01(5)
**c5**	1.3	0.03(6)	0.97(6)
c6	1.7	0.97(10)	0.03(10)
c7	1.9	0.97(5)	0.03(5)
**c8**	2.1	0.04(5)	0.96(5)
c9	3.0	0.93(9)	0.07(9)
c10	3.5	0.91(6)	0.09(6)

a Bolded compositions crystallize in *P*6_2_, while unbolded ones crystallize in the *P*6_4_ enantiomer.

It needs to be mentioned that the SCXRD data refinement of an intermetallic compound consisting of three elements with not-too-different X-ray scattering factors is always a bit ambiguous so that our proposed structural model should be considered as a very probable one, but still might not fully capture the complexity of the structure. For instance, due to the similar metallic radii of Zn, Co, and Mn, mixed occupancy of all three metals at a particular site cannot be ruled out based on the XRD data.

### 2.3 Structure Description

**Figure 1:**
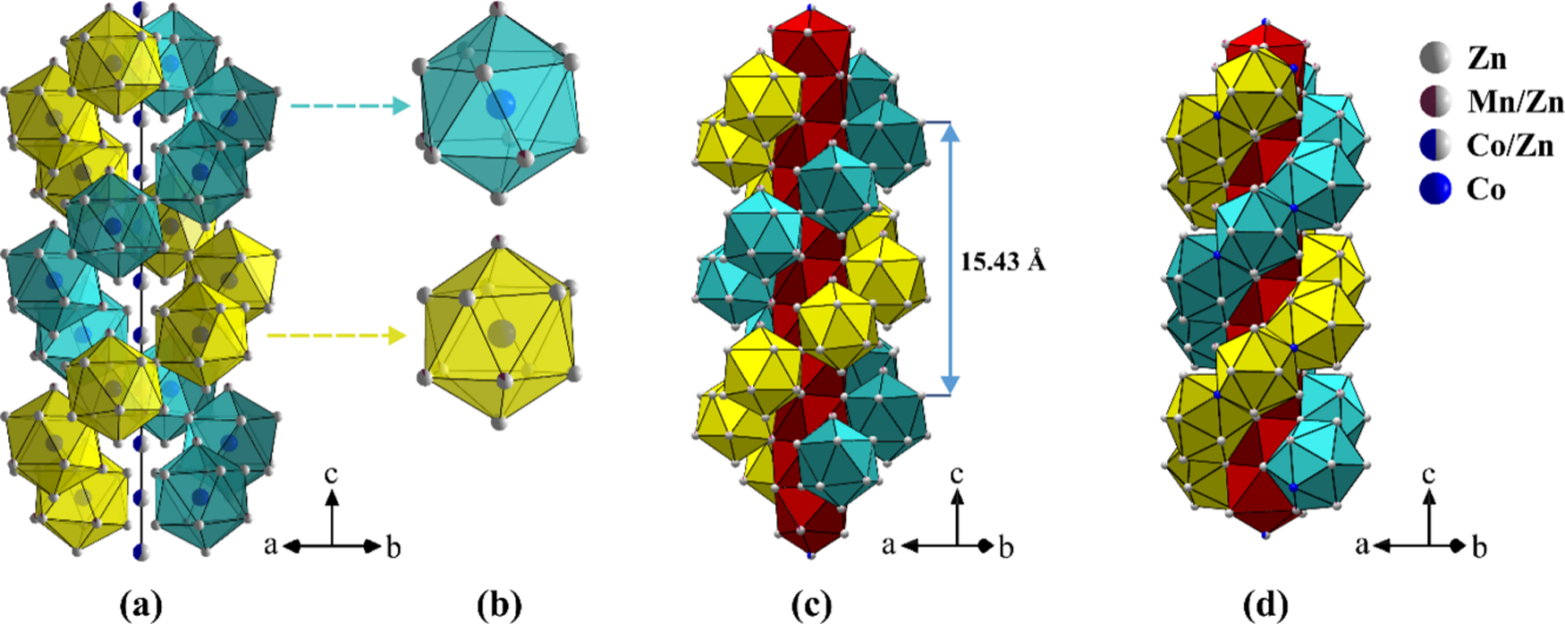
Crystal structure of the δ-Co_2.5_Zn_17.5–*x*_Mn_*x*_ pseudo-binary phase for the c2 composition (loading composition Co_2.5_Zn_16.9_Mn_0.6_). (a) A representation where the double helix of vertex-sharing Co-centered icosahedra wraps around the vertical stand (the 6-fold axis of the structure) made of the Zn8/Co8 3*a* mixed site. (b) Two equivalent icosahedra centered on the Co11 site, one from each helix. (c) The same structure as in (a), where the double helix wraps around a vertical succession of distorted Archimedean antiprisms centered on the Zn8/Co8 sites. (d) An alternative representation of the structure with a double helix of face-sharing icosahedra that wraps around the hub of Archimedean antiprisms.

Below we describe the structure of the c2 composition (the structures of other compositions with the *P*6_2_ symmetry are qualitatively the same, except for the Zn/Mn mixed sites, while the structures of the *P*6_4_ enantiomorphs are mirror images, i.e., left-handed versus right-handed structures). The structure is similar to that of the parent δ-Co_2.5_Zn_17.5_ phase.^7^ The hexagonal unit cell contains 60 atoms and the structure is best visualized by drawing polyhedra around each M11 (6*c*) site that is fully occupied by Co atoms (the Co11 site). These polyhedra are regular icosahedra (coordination number 12), where each icosahedron is comprised of the following atoms: Zn1, Zn2 (×2), Zn3 (×2), Zn4/Mn4, Zn5, Zn6/Mn6 (×2), Zn7, Zn9/Mn9, and Zn10. By sharing vertices, the icosahedra form a double helix wrapped around the vertical stand, which is made of the M8 (Zn8/Co8) 3*a* mixed site and represents the 6-fold axis of the structure (directed along the *c* crystallographic direction). The two helices are crystallographically equivalent, having the same handedness and extending infinitely long along the hexagonal axis. The three Zn/Mn mixed sites are located on the icosahedra. The structure is depicted schematically in Figure 1a, where the two helices are drawn in different colors, although they are equivalent. Two equivalent icosahedra centered on the Co11 site, one from each helix, are shown enlarged in Figure 1b. In the double helix structure, the spiral coil’s distance (or the distance between two Co11 atoms making a complete turn in each helix) is about 15.43 Å, and within this distance, it contains exactly 7 such regular icosahedra, which implies that the unwind number is 7. The width of the double helix is 8.45 Å. The structure can be further visualized by considering that the inside of the double helix forms a succession of distorted Archimedean antiprisms, each one centered at the Zn8/Co8 mixed-occupied 3*a* position. The double helix then wraps around the hub of Archimedean antiprisms, as shown in Figure 1c.

An alternative description of the structure can be made by taking the shared icosahedra vertices as the centers of icosahedra that share faces to form a regular double helix of icosahedra, as defined by Lidin and Andersson.^16^ The inside of the double helix again forms a succession of distorted Archimedean antiprisms centered at the Zn8/Co8 mixed-occupied 3*a* position. The double helix of face-sharing icosahedra then wraps around the hub of Archimedean antiprisms, as shown in Figure 1d.

### 2.4 Mn Substitution Pattern

The Mn substitution pattern in the δ-Co_2.5_Zn_17.5–*x*_Mn_*x*_ (*x* = 0.4–3.5) pseudo-binary alloys was studied by SCXRD structure refinement for a set of 10 compositions c1 to c10 with a systematically increasing Mn content. The refined (final) compositions in atom % are ranging approximately from Co_12_Zn_86_Mn_2_ (c1) to Co_12_Zn_71_Mn_17_ (c10). The results are presented in Table 5, where the site occupancy of Mn at the Wyckoff sites M3, M4, M5, M6, M9, and M10 (all in 6*c*) is given for the 10 investigated compositions.

**Table 5:** Mn Site Occupancy at the Zn/Mn Mixed Wyckoff Sites M3, M4, M5, M6, M9, and M10 (all in 6*c*) for the 10 Investigated Compositions c1 to c10

refined composition (atom %)	M3	M4	M5	M6	M9	M10
Co_11.99_Zn_85.97_Mn_2.04_ (c1)	0	0.04(5)	0	0.04(5)	0.12(4)	0
Co_12.17_ Zn_84.67_Mn_3.16_ (c2)	0	0.092(12)	0	0.070(10)	0.154(9)	0
Co_12.24_ Zn_82.72_Mn_5.04_ (c3)	0	0.113(13)	0.082(10)	0.085(11)	0.225(9)	0
Co_12.22_Zn_82.14_Mn_5.64_ (c4)	0	0.139(12)	0.134(9)	0.087(10)	0.204(9)	0
Co_11.99_Zn_81.22_Mn_6.79_ (c5)	0	0.115(13)	0.155(11)	0.136(12)	0.273(11)	0
Co_12.09_Zn_80.12_Mn_7.79_ (c6)	0	0.17(2)	0.225(19)	0.13(2)	0.257(19)	0
Co_12.06_Zn_78.42_Mn_9.52_ (c7)	0.044(9)	0.133(13)	0.249(11)	0.223(11)	0.303(11)	0
Co_11.39_Zn_78.07_Mn_10.54_ (c8)	0.049(7)	0.148(11)	0.271(9)	0.201(10)	0.385(9)	0
Co_12.03_Zn_72.41_Mn_15.56_ (c9)	0.150(14)	0.23(2)	0.386(18)	0.316(19)	0.402(18)	0.075(18)
Co_11.76_Zn_70.87_Mn_17.37_ (c10)	0.157(8)	0.167(13)	0.422(11)	0.407(12)	0.454(11)	0.130(11)

**Figure 2:**
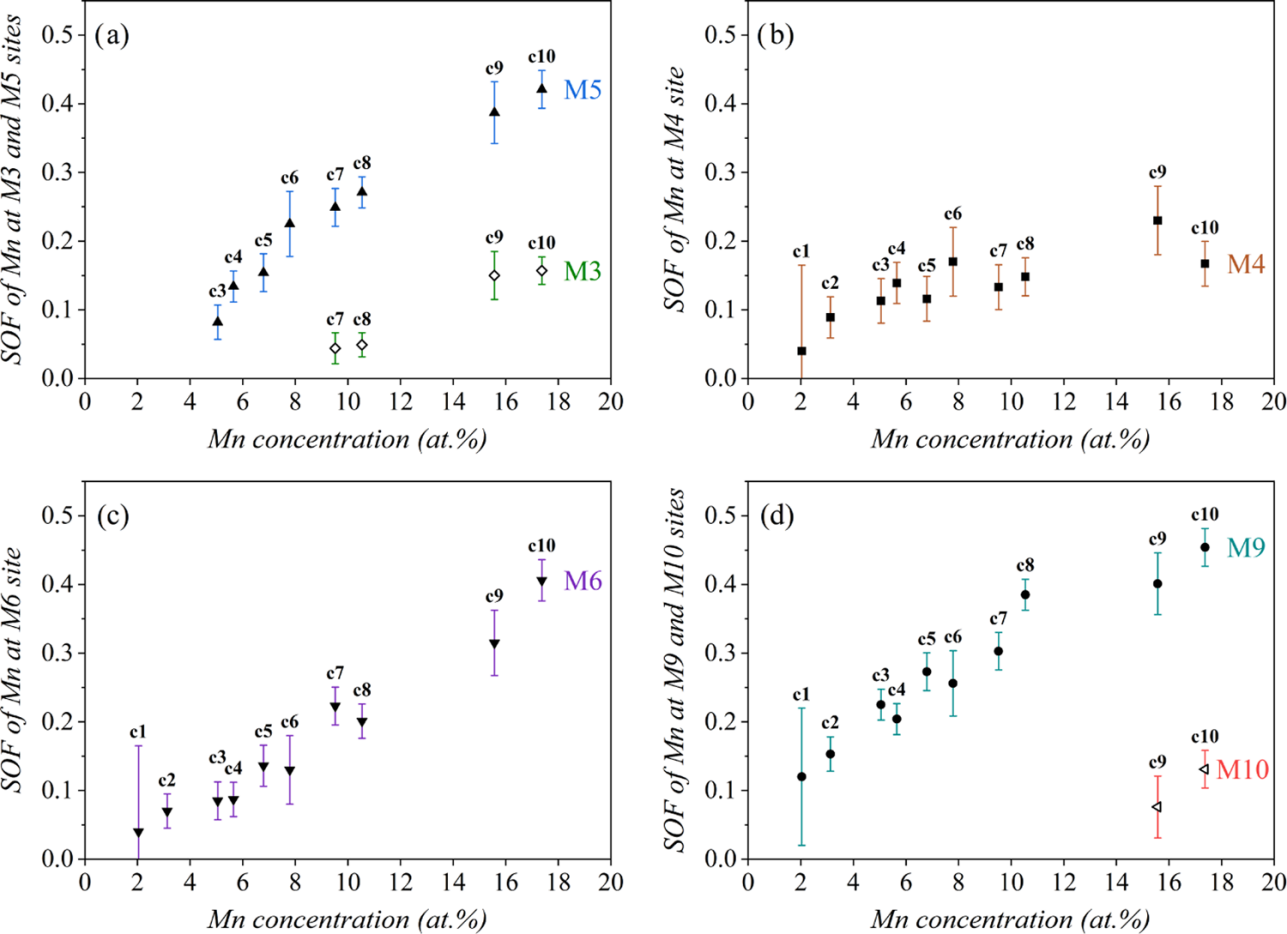
Site occupancy factors (SOF) of Mn at the Zn/Mn mixed Wyckoff sites (a) M3 and M5, (b) M4, (c) M6, and (d) M9 and M10 for the 10 investigated compositions c1 to c10 (with reference to Table 5). The Mn concentrations (in atom %) on the abscissae are taken from the refined compositions given in Table 5.

For the two compositions with the lowest Mn contents (c1 and c2, having the refined Mn concentrations of 2.04 and 3.16 atom %, respectively), Mn partially substitutes Zn at three sites M4, M6, and M9. At intermediate Mn contents (compositions c3 to c6, with the Mn concentrations ranging from 5.04 to 7.79 atom %), the M5 site becomes additionally Zn/Mn mixed populated. At high Mn contents (c7 to c10, Mn concentrations from 9.52 to 17.37 atom %), Mn partially replaces Zn also at the M3 site, and for the two highest Mn-containing compositions c9 and c10, also at the M10 site, so that there are six Zn/Mn mixed-populated sites (M3, M4, M5, M6, M9, and M10). The Mn occupations of these sites increase systematically with the increasing Mn content in the alloys, as shown in Figure 2. For the composition c10 with the highest Mn content, the Mn occupancies of the mixed sites (rounded to two digits) are M3:0.16, M4:0.17, M5:0.42, M6:0.41, M9:0.45, and M10:0.13. All six Zn/Mn mixed sites are located on the icosahedra.

### 2.5 Phase Analysis by Powder X-ray Diffraction

**Figure 3:**
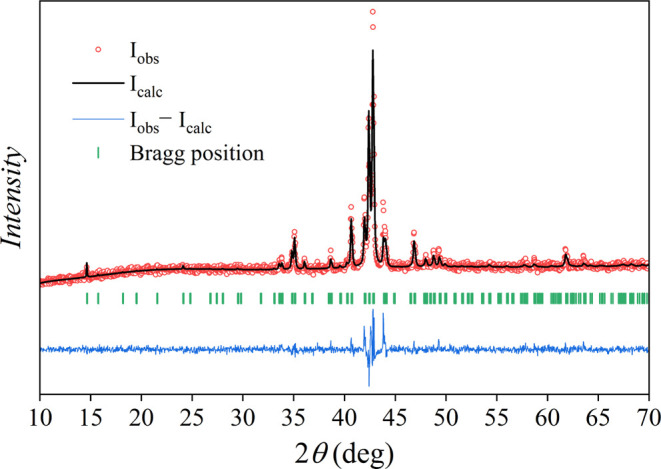
Rietveld refinement plot for the powder sample of c2 composition (loading composition Co_2.5_Zn_16.9_Mn_0.6_) with the refinement parameters *R*(obs) *=* 5.30%, *w*R**(obs) = 5.12%, *R*_p_ = 5.16%, *w*R**_p_ = 6.82%, GOF = 1.35. *I*_obs_ is the observed intensity, *I*_calc_ is the calculated intensity, and *I*_obs_ – *I*_calc_ is their difference. Bragg positions are shown as stick spectra.

Powder X-ray diffraction (PXRD) was used to investigate the phase purity of the 10 investigated δ-Co_2.5_Zn_17.5–*x*_Mn_*x*_ compositions, by performing Rietveld refinement. The structural model determined before by SCXRD was imported and compared to the experimental powder diffraction patterns. The three main parameters (*R*(*F*), GOF, and *w*R**(*F*)) were monitored. The Rietveld refinement plot for the c2 composition is shown in Figure 3, with the values of GOF and other refinement parameters given in the figure caption. It is observed that all experimental peaks match well with the simulated peaks, indicating phase purity of the sample, i.e., only the δ-phase is present. The Rietveld refinement plots for other compositions are not shown but are qualitatively similar to that of the c2 composition. For the compositions with Mn content beyond *x* ≈ 3.5, the cubic γ-brass type phase (*I*4̅_3_*m*) starts to form. According to this, we conclude that the homogeneity range of the δ-phase chiral structure (either *P*6_2_ or *P*6_4_) in the Co_2.5_Zn_17.5–*x*_Mn_*x*_ pseudo-binary alloys is within the range *x* ≈ 0–3.5.

## 3 Magnetism of the *x* = 0.4 Compound

A detailed magnetic study of the δ-Co_2.5_Zn_17.5–*x*_Mn_*x*_ pseudo-binary alloys was conducted for the lowest Mn-containing composition c1 (loading composition Co_2.5_Zn_17.1_Mn_0.4_, or Co_12.5_Zn_85.5_Mn_2.0_, in atom %). In the following, we shall use an abbreviated denotation of this sample as *x* = 0.4. Its chemical composition was verified by scanning electron microscopy (SEM) energy-dispersive X-ray spectroscopy (EDS). The EDS composition averaged over 10 measured points within the area of 50 × 50 μm^2^, and rounded to the first integers was Co_11_Zn_87_Mn_2_ (with the experimental uncertainty of at least 1 atom %). According to the Mn substitution pattern presented in Table 5, the c1 composition has three Zn/Mn mixed sites (Zn4/Mn4, Zn6/Mn6, and Zn9/Mn9). The magnetic lattice consists of the Co and Mn magnetic ions. The Co sublattice is identical to that of the δ-Co_2.5_Zn_17.5_ parent compound (shown in Figure S1b in the Supporting Information), where the Co atoms from the icosahedra centers form a regular double helix structure (with all sites fully occupied), while the Co atoms on the 6-axis (the mixed Zn8/Co8 site with the Zn/Co occupancies^7^ 0.58/0.42) are statistically randomly substituted by the nonmagnetic Zn, approximately half–half, introducing some randomness into the magnetic lattice. The Mn sublattice with the three Zn/Mn mixed-occupied sites on the icosahedra introduces additional randomness into the magnetic lattice of the *x* = 0.4 alloy because the magnetic Mn and nonmagnetic Zn statistically substitute each other. A significant fraction of the Co moments and all Mn moments are hence randomly positioned in the crystal structure. Due to the metallic character of the alloy, it is reasonable to assume that the interactions between the magnetic moments are of the indirect-exchange (Ruderman–Kittel–Kasuya–Yosida – RKKY) type. Since there are two kinds of magnetic moments (Co and Mn) in the system, there will be three kinds of RKKY couplings present, the Co–Co, Mn–Mn, and Co–Mn, where each of them may favor either parallel or antiparallel spin alignment. The sign of the RKKY coupling also fluctuates between positive and negative values with the interspin distance on the scale of nanometers. All this suggests the formation of a magnetically frustrated state.

### 3.1 Direct-Current Magnetic Susceptibility

**Figure 4:**
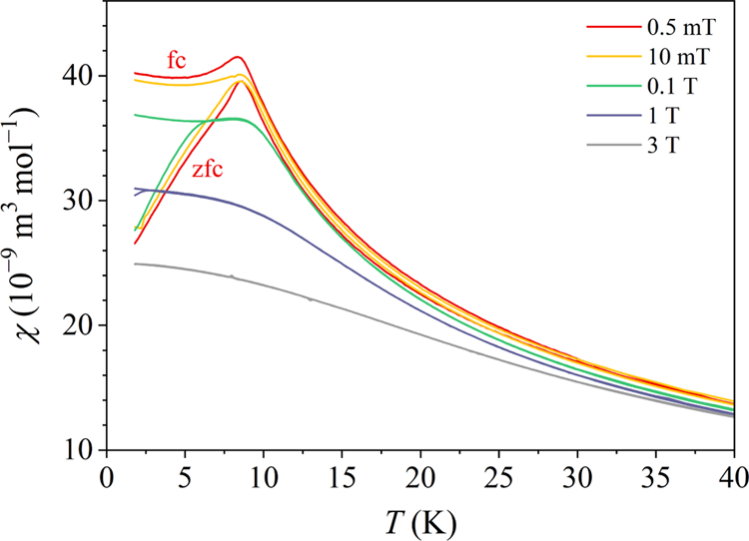
Zfc and fc dc magnetic susceptibilities χ = *M*/*H* of the *x* = 0.4 alloy in the temperature range between 40 and 1.8 K in a set of magnetic fields from μ_0_*H* = 0.5 mT to 3 T.

The direct-current (dc) magnetic susceptibility was measured in the temperature range between 300 and 1.8 K in a set of magnetic fields ranging from μ_0_*H* = 0.5 mT to 7 T. The measurements were performed for both zero-field-cooled (zfc) and field-cooled (fc) protocols. The magnetization divided by the magnetic field (the susceptibility) χ = *M*/*H* in the temperature range below 40 K in magnetic fields μ_0_*H* = 0.5, 10 mT, 0.1, 1 and 3 T is shown in Figure 4.

In the two lowest fields (0.5 and 10 mT), the zfc and fc susceptibilities χ_zfc_ = *M*_zfc_/*H* and χ_fc_ = *M*_fc_/*H* are more or less identical at temperatures above 8 K (a tiny splitting can be noticed also above 8 K, indicating the presence of some small spin clusters that magnetically order already at slightly higher temperatures), while they start to differ significantly below that temperature. χ_fc_ remains roughly constant, whereas χ_zfc_ exhibits a cusp (a maximum) and then decreases toward zero upon cooling. Such behavior is characteristic of magnetically frustrated spin systems with broken ergodicity at low temperatures.^17^ The temperature where χ_zfc_ exhibits a cusp is conveniently defined as the spin freezing temperature, amounting to *T*_f_ ≈ 8 K in this case. The spin freezing temperature is considered as the temperature below which thermal spin fluctuations become so slow that the system cannot reach thermal equilibrium anymore on the accessible experimental time scale. The ergodicity of the spin system is consequently broken and the experimentally observable physical quantities become time-dependent, depending on the observation time window of a given experimental technique. In higher magnetic fields (at μ_0_*H* ≥ 0.1 T), the χ_fc_ – χ_zfc_ bifurcation temperature shifts continuously to lower temperatures, until in the field of 3 T the difference between χ_fc_ and χ_zfc_ is no more observed down to the lowest investigated temperature of 1.8 K. The magnetic field of this magnitude is already strong enough that the Zeeman interaction wins over the exchange interaction and destroys the fragile internal magnetic structure of the frustrated state.

### 3.2 Alternating-Current Magnetic Susceptibility

**Figure 5:**
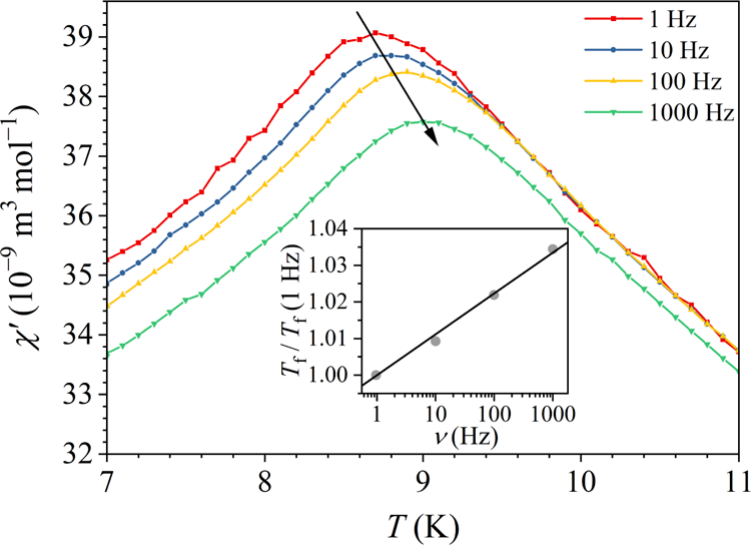
Real part χ′ of the ac magnetic susceptibility at logarithmically spaced frequencies ν between 1 and 1000 Hz in the temperature regime of the ergodic-nonergodic phase transition. The inset shows the (*T*_f_ (ν))/(*T*_f_ (1 Hz)) relation, where the spin freezing temperature *T*_f_ is defined as the temperature of the peak in χ′.

The alternating-current (ac) magnetic susceptibility was measured in a sinusoidal magnetic field of amplitude μ_0_*H*_0_ = 0.2 mT at logarithmically spaced frequencies ν between 1 and 1000 Hz. The real part of the ac susceptibility χ′ in the temperature range of the ergodic-nonergodic phase transition is shown in Figure 5. A frequency-dependent peak in χ′ is observed, which shifts to higher temperatures at higher frequencies. The temperature of the peak is associated with the frequency-dependent spin freezing temperature *T*_f_ (ν), which at the lowest investigated frequency ν = 1 Hz amounts to *T*_f_ (1 Hz) = 8.7 K. The (*T*_f_ (ν))/(*T*_f_ (1 Hz)) relation is presented in the inset of Figure 5. A logarithmic increase of the spin freezing temperature with increasing frequency is evident, demonstrating the dependence of this observable physical parameter on the observation time (or frequency) window of the employed ac susceptibility experimental technique applied to the spin system with broken ergodicity. The fractional shift of the spin freezing temperature per decade of frequency is Γ = Δ*T*_f_/*T*_f_Δ(log ν) = 1.1 × 10^–2^. This value falls into the range of spin glasses, where the values Γ ≈ 10^–2^–10^–3^ are common.^18^

### 3.3 Paramagnetic Susceptibility

At temperatures above the spin freezing temperature (in the paramagnetic regime), the temperature dependence of the susceptibility is of a Curie–Weiss type and it was analyzed by the expression
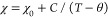
1The second term on the right is the Curie–Weiss susceptibility of localized paramagnetic moments, where *C* is the Curie–Weiss constant, θ is the Curie–Weiss temperature, and χ_0_ is the temperature-independent contribution to the total susceptibility. χ_0_ is a sum of the positive Pauli spin susceptibility χ_P_ of conduction electrons, the negative Larmor susceptibility χ_Larmor_ of closed electronic shells, and the negative Landau susceptibility χ_Landau_ of the conduction electrons due to their orbital circulation in an external magnetic field.

**Figure 6:**
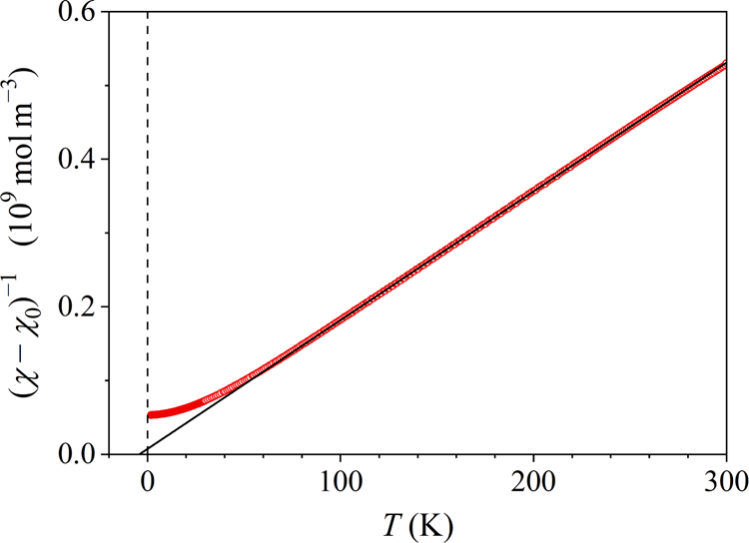
Magnetic susceptibility χ = *M*/*H* measured in the field 7 T in a (χ – χ_0_)^−1^ vs *T* plot. The solid line is the Curie–Weiss fit in the paramagnetic regime (the fit parameter values are given in the text).

The temperature-dependent susceptibility χ(*T*) measured in the magnetic field μ_0_*H* = 7 T was analyzed with eq 1 in the paramagnetic regime at temperatures between 50 and 300 K. In Figure 6, the susceptibility is presented in a (χ – χ_0_)^−1^ vs *T* plot and the fit is shown by a solid line. The fit parameters are *C* = 5.7 × 10^–7^ m^3^K mol^–1^, θ = −4 K and χ_0_ = 1.7 × 10^–10^ m^3^ mol^–1^. The small negative Curie–Weiss temperature indicates that the average spin coupling is slightly antiferromagnetic (AFM). The Curie–Weiss constant allows calculating the mean effective paramagnetic moment μ̅_eff_ = *p̅*_eff_μ_*B*_ (where *p̅*_eff_ is the mean effective Bohr magneton number and μ_B_ is the Bohr magneton) of the combined Co–Mn spin system from the formula ,^19^ yielding μ̅_eff_ = 0.60 μ_B_. This value is strongly reduced with regard to the experimental paramagnetic free-ion values of the Mn^3+^, Mn^2+^, Co^3+^, and Co^2+^ ions that amount to 4.9, 5.9, 5.4, and 4.8 μ_B_, respectively. Such reduction of the moment in an electrically conducting environment is common. The positive χ_0_ value indicates that the Pauli spin susceptibility of the conduction electrons dominates the temperature-independent term in the total susceptibility. Within the free-electron model, the three contributions to χ_0_ (χ_P_, |χ_Larmor_| and |χ_Landau_|) are of the same order of magnitude. An estimate of the Larmor contribution from literature tables^20^ has yielded χ_Larmor_ ≈ – 1.8 × 10^–10^ m^3^ mol^–1^, which is of the correct order of magnitude in comparison to the fit-determined χ_0_ value.

### 3.4 *M*(*H*) Magnetization Curves

**Figure 7:**
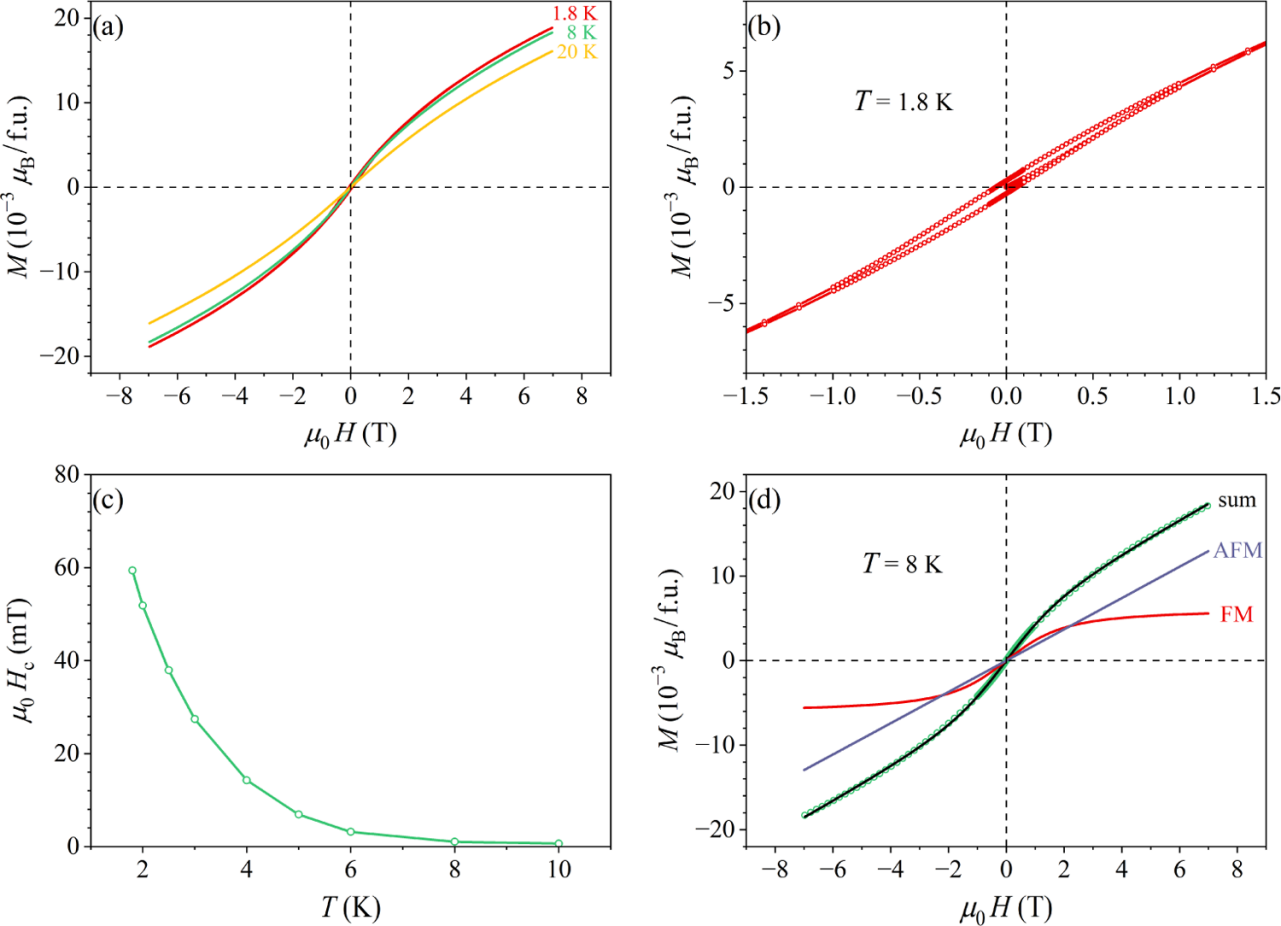
(a) *M*(*H*) curves at temperatures 20, 8, and 1.8 K, with the magnetization given in units of μ_B_ per formula unit (f.u.), i.e., per one Co_0.11_Zn_0.87_Mn_0.02_ average “atom”. (b) Hysteresis of the 1.8 K curve on an expanded scale. (c) Temperature-dependent coercive field *H*_c_. (d) Theoretical fit of the 8 K *M*(*H*) curve with eq 2 (the values of the fit parameters are given in the text). The FM and AFM contributions to the total magnetization are also shown separately.

The magnetization versus the magnetic field, *M*(*H*), curves were determined for the field sweep μ_0_*H* = ± 7 T. The *M*(*H*) curves at low temperatures (*T* = 20, 8, and 1.8 K), with the magnetization given in units of μ_B_ per formula unit (f.u.), i.e., per one Co_0.11_Zn_0.87_Mn_0.02_ average “atom”, are shown in Figure 7a. Hysteresis appears below 8 K (within the nonergodic phase) and the coercivity *H*_c_ increases with decreasing temperature. The hysteresis of the 1.8 K curve on an expanded scale is shown in Figure 7b. The hysteresis loop is narrow, with the coercive field of μ_0_*H*_c_ ≈ 60 mT, and closes up in the field of about 1.5 T, which is typical for the AFM-type hysteresis (the FM-type hysteresis loops typically close in a much lower field of about 0.2 T). The temperature-dependent coercive field *H*_c_ is presented in Figure 7c.

**Figure 8:**
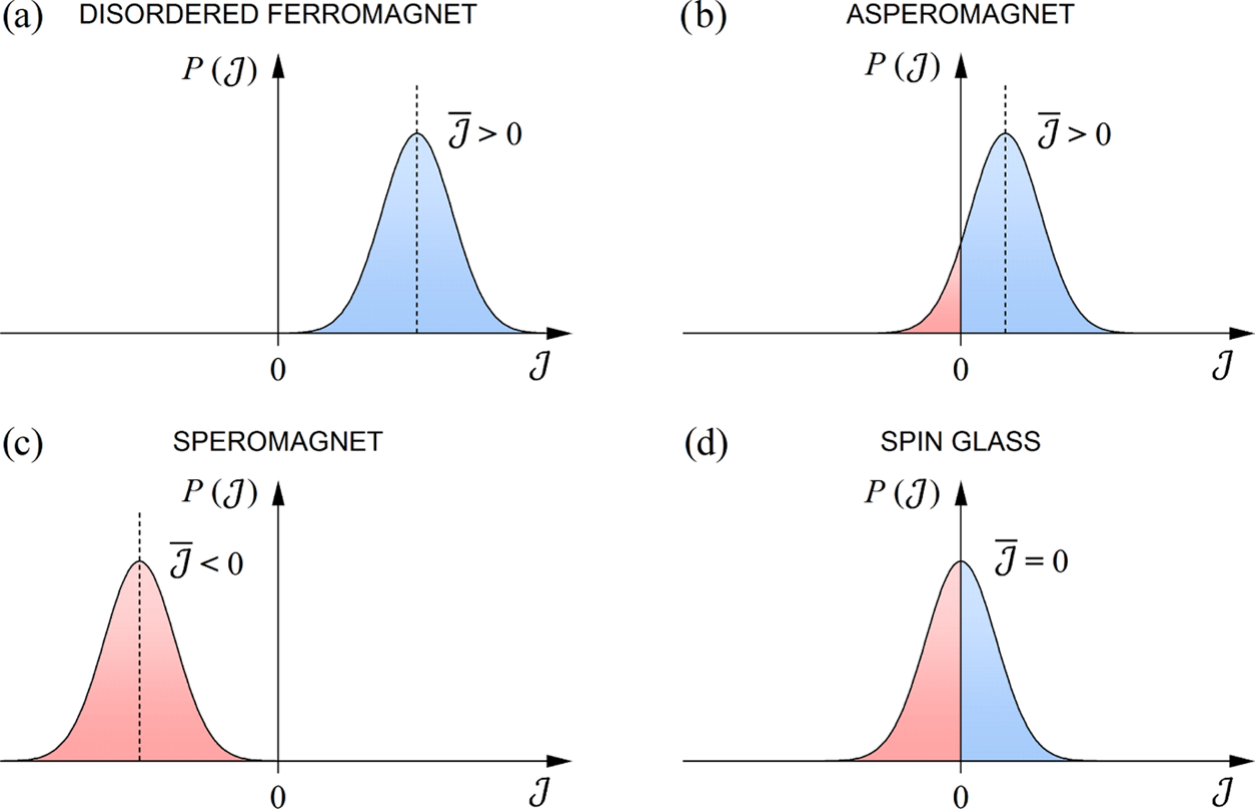
Schematic presentation of the distribution function of the exchange coupling constants  that determines the type of magnetic order in exchange-dominated disordered magnetic systems: (a) a disordered ferromagnet, (b) an asperomagnet, (c) a speromagnet, and (d) a spin glass.

The *M*(*H*) curve at *T* = 8 K, where the hysteresis is still absent, was theoretically analyzed by assuming that the investigated δ-Co_2.5_Zn_17.5–*x*_Mn_*x*_ alloys are exchange-dominated spin systems, since in chemically disordered 3*d* magnetic alloys, random local magnetic anisotropy at the atomic scale is generally insufficient to pin the local magnetization direction. In a disordered spin system with mixed FM-AFM interactions, the exchange coupling constant  is distributed and can be modeled by a continuous, symmetric bell-like distribution function  that extends on both  (FM) and  (AFM) sides of the  axis.^21^ The distribution  for different types of disordered magnetic systems (a disordered ferromagnet, an asperomagnet, a speromagnet, and a spin glass, where the asperomagnetic and speromagnetic states are known in the context of amorphous magnets^21^) is shown schematically in Figure 8. These magnetic states differ in the position of the maximum of  on the  axis that peaks at the average exchange coupling constant  (, , or ).

The shape of the *M*(*H*) curve was modeled by a function
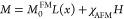
2Here, the term *M*_0_^FM^*L*(*x*) describes the FM contribution to the total magnetization *M*, accounting for the part of  on the  (FM) side of the  axis. *M*_0_^FM^ is the saturated FM magnetization, whereas *L*(*x*) with *x* = μμ_0_*H*/*k*_*B*_*T* is the Langevin function, in which the magnetic moment μ is treated as a classical vector that can assume any value (μ → ∞), accounting for the large effective FM group moments. The term χ_AFM_*H* describes the AFM contribution to the magnetization (with χ_AFM_ denoting the AFM susceptibility), accounting for the part of  on the  (AFM) side of the  axis. The FM and AFM magnetization contributions show different asymptotic behavior at large magnetic field *H*. While the FM part saturates to a constant (horizontal) plateau determined by *M*_0_^FM^, the AFM part remains linear in *H* at any experimentally accessible field. The total magnetization *M*(*H*) curve consequently shows initial rapid increase at low fields (lead by the Langevin function), which becomes slower at moderate fields, while at large *H*, *M* approaches asymptotically an inclined straight line with the slope χ_AFM_. This behavior is different from classical ferromagnets, where *M* saturates to a horizontal plateau at large *H*, while for classical antiferromagnets it is linear in *H* at any magnetic field.

The theoretical fit of the 8 K *M*(*H*) curve with eq 2 is shown in Figure 7d. The FM and AFM contributions to the total magnetization are also shown separately. The values of the fit parameters are *M*_0_^FM^ = 6.3 × 10^–3^ μ_B_/f.u., μ = 15.1 μ_B_ (indicating quite small average size of the FM clusters) and χ_AFM_/μ_0_ = 1.85 × 10^–3^ μ_B_/(f.u.T). The fit proves the existence of mixed FM-AFM interactions.

### 3.5 Thermoremanent Magnetization

An important physical quantity to characterize spin systems with broken ergodicity is the time decay of the thermoremanent magnetization (TRM), which is logarithmically slow and proceeds with time constants of hours, days, or years, depending on temperature. The reason for the ultraslow time decay is a broad, continuous distribution of spin fluctuation times in a magnetically frustrated system, which extends from microscopic up to macroscopic times. The TRM time-decay measurement protocol involves continuous cooling of the spin system in a magnetic field *H*_fc_ (with “fc” denoting field-cooling) from the paramagnetic phase through the spin freezing temperature *T*_f_ into the nonergodic phase, where the cooling is stopped at a certain measuring (aging) temperature *T*_m_ and the spin system is left to age in *H*_fc_ isothermally for a waiting (aging) time *t*_w_ of the order of minutes to hours. At the end of the aging period (after *t*_w_), the magnetization of the frustrated spin system is *M*_fc_. In the next step, the magnetic field *H*_fc_ is suddenly cut to zero. The reversible part of the magnetization *M*_rev_ vanishes with the field instantaneously, whereas the thermoremanent magnetization *M*_TRM_ decays in time *t* logarithmically slow after the field cut-off. This decay is experimentally monitored over macroscopic times, typically over several hours. The TRM time decay *M*_TRM_ (*t*) takes place in zero magnetic field and describes slow approach of the spin system toward the thermal equilibrium state with zero magnetization, but the global equilibrium can never be reached since the equilibration times are much longer than the observation time window of the TRM experiment, resulting in broken ergodicity of the spin system. The TRM decay depends on aging temperature *T*_m_, aging time *t*_w_, and cooling field *H*_fc_, where the TRM amplitude at the beginning of the decay normalized to the magnetization just before the field cut-off, *M*_TRM_ (*t* = 0)/*M*_fc_, increases with decreasing *T*_m_ and increasing *t*_w_ but decreases with increasing *H*_fc_. The shape of the TRM time-decay curve, *M*_TRM_ (*t*)/*M*_fc_, also depends on these three parameters, where the decay generally slows down at lower *T*_m_ and for longer *t*_w_, whereas it becomes faster for increasing *H*_fc_. This behavior is difficult to treat theoretically because it represents the out-of-equilibrium dynamics of a nonergodic spin system. An explanation has been proposed for spin glasses,^17,22−30^ which assumes highly structured phase space of the collective (exchange-coupled) spin system that contains a plethora of degenerate global and local minima separated by a hierarchical distribution of exchange barriers. Upon searching for the global equilibrium, the spin system explores different metastable states in the phase space by thermally activated overbarrier hopping, but the process is so slow that only a small part of the phase space is visited within the observation time window of the employed experimental technique. The TRM time decay hence represents the slow approach of the frustrated spin system toward the thermal equilibrium state, which can never be reached on the available experimental time scale.

**Figure 9:**
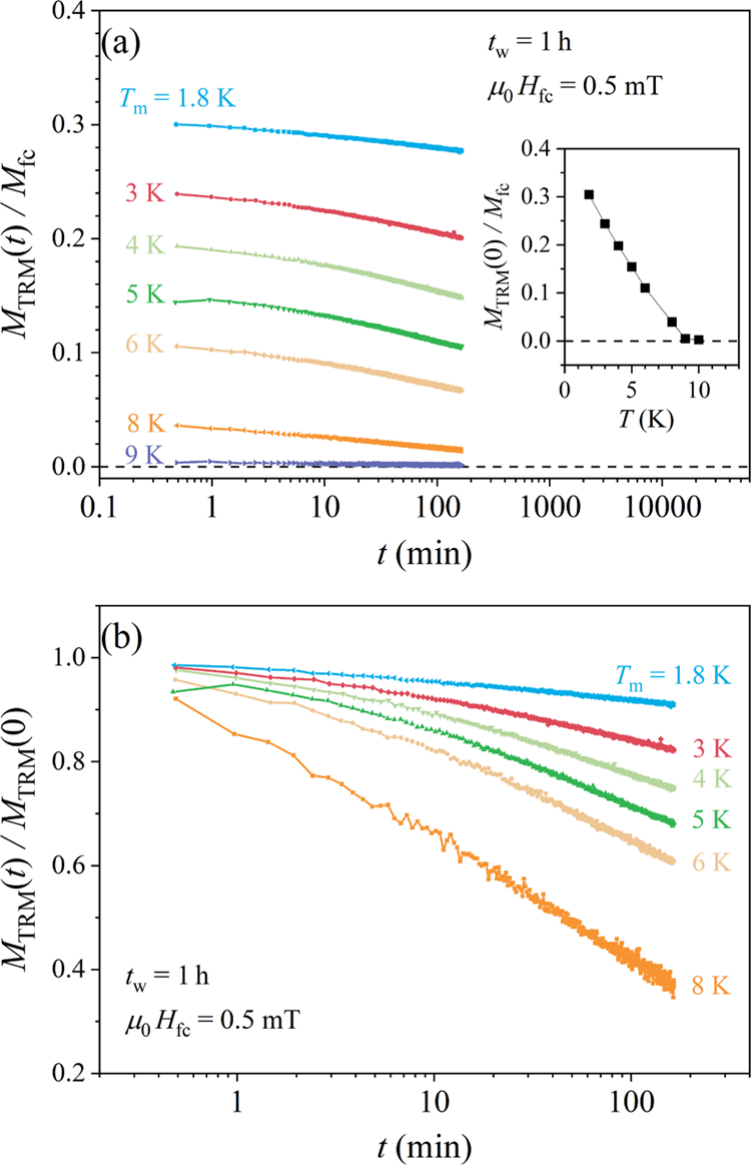
(a) TRM time-decay curves *M*_TRM_ (*T*_m_,*t*)/*M*_fc_ (*T*_m_) at the measuring temperatures *T*_m_ between 9 and 1.8 K for a waiting time *t*_w_ = 1 h. The inset shows the normalized amplitude at the beginning of the decay, *M*_TRM_ (*T*_m_,*t* = 0)/*M*_fc_ (*T*_m_), as a function of *T*_m_. (b) The same TRM decay curves normalized to their *t* = 0 values, *M*_TRM_ (*T*_m_,*t*)/*M*_TRM_ (*T*_m_,*t* = 0).

The TRM time-decays of the *x* = 0.4 alloy for a set of measuring temperatures *T*_m_ between 9 and 1.8 K are shown in Figure 9. A small cooling field μ_0_*H* = 0.5 mT was applied and the material was cooled continuously in that field from the paramagnetic phase to the measuring temperature *T*_m_, where aging for a time *t*_w_ = 1 h was employed. After the field cut-off, the TRM decays were monitored for a time of approximately 3 h. The normalized TRM decay curves *M*_TRM_ (*T*_m_,*t*)/*M*_fc_ (*T*_m_) are shown in Figure 9a, whereas the TRM amplitude at the beginning of the decay, *M*_TRM_ (*T*_m_,*t* = 0)/*M*_fc_ (*T*_m_), as a function of *T*_m_ is shown in the inset. Within the nonergodic phase (below *T*_f_ ≈ 8 K), a systematic increase of the TRM amplitude with decreasing *T*_m_ is evident, whereas there is no TRM in the ergodic phase above 8 K. The TRM time-decays also slow down with the decreasing *T*_m_ (the remanence of the spin system increases on cooling), which is demonstrated in Figure 9b, where the TRM decay curves are shown normalized to their *t* = 0 values, *M*_TRM_ (*T*_m_,*t*)/*M*_TRM_ (*T*_m_,*t* = 0).

**Figure 10:**
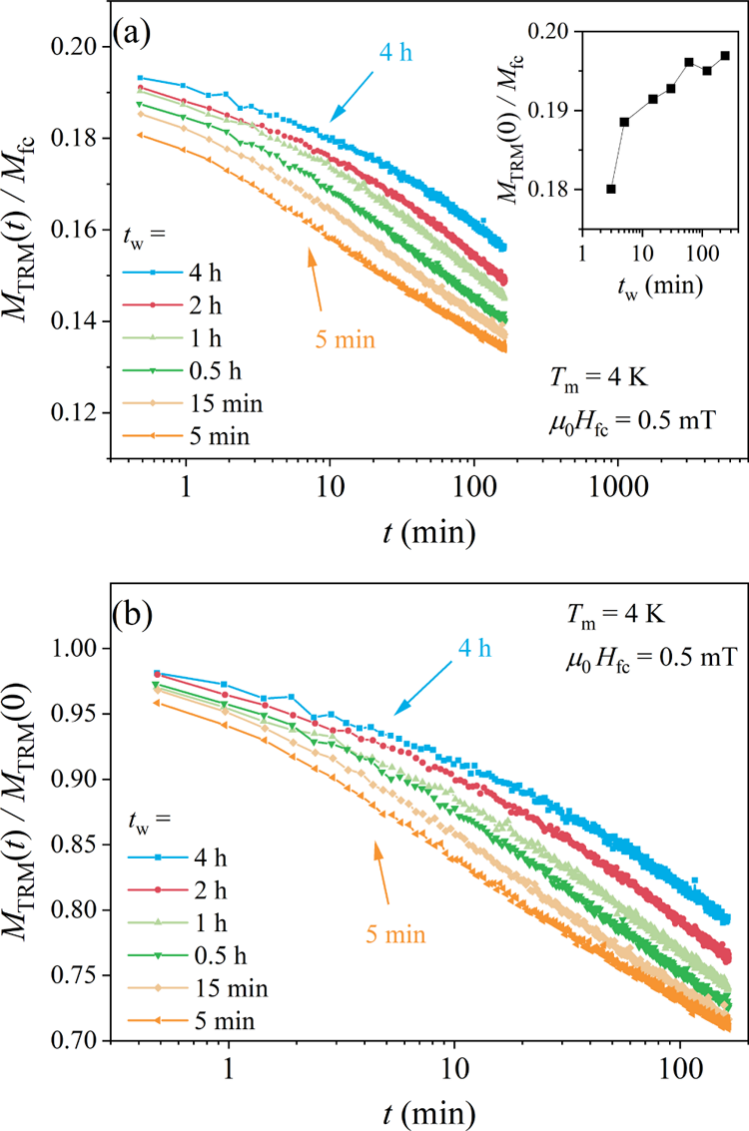
(a) TRM time-decay curves *M*_TRM_ (*t*_w_,*t*)/*M*_*fc*_ (*t*_w_) for a set of aging times *t*_w_ between 5 min and 4 h at the measuring temperature *T*_m_ = 4 K. The inset shows the normalized amplitude at the beginning of the decay, *M*_TRM_ (*t*_w_,*t* = 0)/*M*_fc_ (*t*_w_), as a function of *t*_w_. (b) The same TRM decay curves normalized to their *t* = 0 values, *M*_TRM_ (*t*_w_,*t*)/*M*_TRM_ (*t*_w_,*t* = 0).

The TRM time-decays as a function of the aging time *t*_w_ at the measuring temperature *T*_m_ = 4 K, obtained after cooling in the field μ_0_*H* = 0.5 mT are shown in Figure 10 for a set of aging times between 5 min and 4 h, approximately logarithmically spaced. In Figure 10a, the normalized decay curves *M*_TRM_ (*t*_w_,*t*)/*M*_fc_ (*t*_w_) are shown, whereas the TRM amplitude at the beginning of the decay, *M*_TRM_ (*t*_w_,*t* = 0)/*M*_fc_ (*t*_w_), as a function of *t*_w_ is shown in the inset. An increased remanence with increasing *t*_w_ is clearly evident. The time-decays also slow down with the increasing *t*_w_, which is demonstrated in Figure 10b, where the TRM decay curves are presented normalized to their *t* = 0 values, *M*_TRM_ (*t*_w_,*t*)/*M*_TRM_ (*t*_w_,*t* = 0).

**Figure 11:**
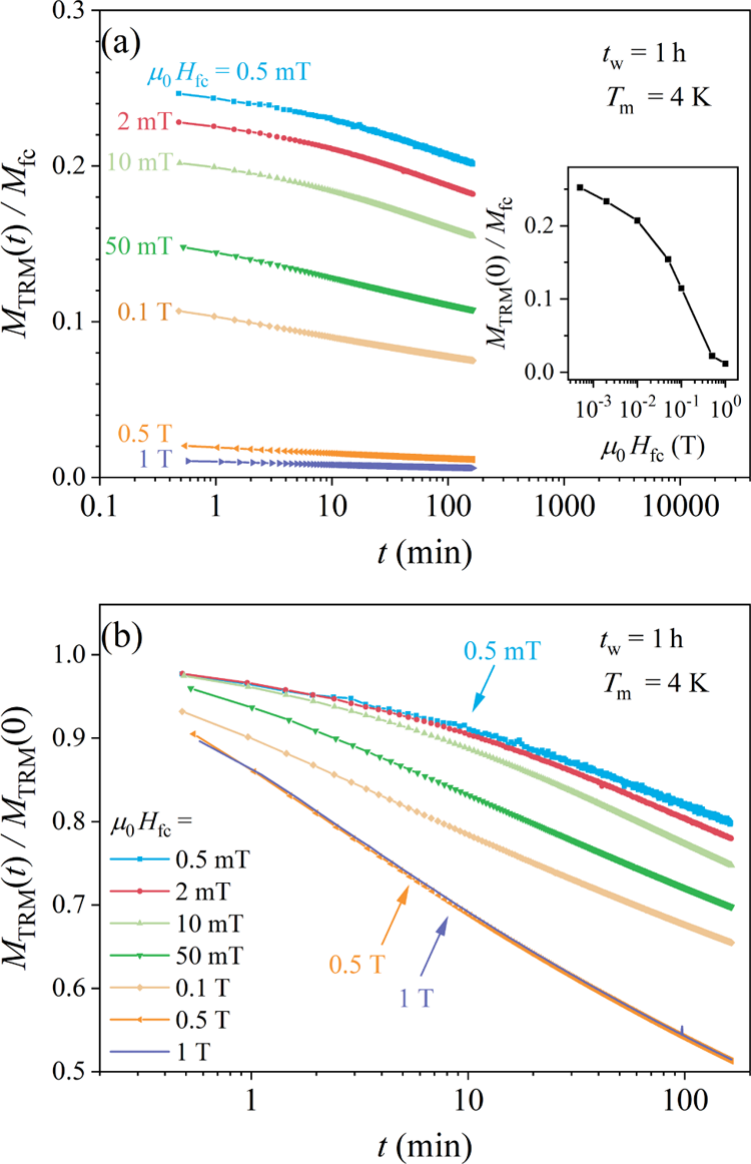
(a) TRM time-decay curves *M*_TRM_ (*H*_fc_,*t*)/*M*_fc_ (*H*_fc_) for a set of cooling fields μ_0_*H*_fc_ between 0.5 mT and 1 T at the measuring temperature *T*_m_ = 4 K for the aging time *t*_w_ = 1 h. The inset shows the normalized amplitude at the beginning of the decay, *M*_TRM_ (*H*_fc_,*t* = 0)/*M*_fc_ (*H*_fc_), as a function of *H*_fc_. (b) The same TRM decay curves normalized to their *t* = 0 values, *M*_TRM_ (*H*_fc_,*t*)/*M*_TRM_ (*H*_fc_,*t* = 0).

The TRM time-decays as a function of the cooling field *H*_fc_, at the measuring temperature *T*_m_ = 4 K and for the aging time *t*_w_ = 1 h are shown in Figure 11 for a set of magnetic fields μ_0_*H*_fc_ between 0.5 mT and 1 T, approximately logarithmically spaced. In Figure 11a, the normalized decay curves *M*_TRM_ (*H*_fc_,*t*)/*M*_fc_ (*H*_fc_) are shown, whereas the TRM amplitude at the beginning of the decay, *M*_TRM_ (*H*_fc_,*t* = 0)/*M*_*fc*_ (*H*_fc_), as a function of *H*_fc_ is shown in the inset. A strong decrease of the TRM with the increasing *H*_fc_ by more than 1 order of magnitude is evident. The shape of the time-decays also changes with *H*_fc_, which is demonstrated in Figure 11b, where the TRM decay curves are presented normalized to their *t* = 0 values, *M*_TRM_ (*H*_fc_,*t*)/*M*_TRM_ (*H*_fc_,*t* = 0). The decays show a tendency to speed up for increasing *H*_fc_.

### 3.6 Memory Effect

The memory effect (ME) is a spectacular manifestation of the ultraslow out-of-equilibrium dynamics of a nonergodic frustrated spin system, which remembers its cooling history within the nonergodic phase that has happened in the absence of an external magnetic field.^17,22,24−33^ The ME measurement protocol involves continuous cooling of the spin system in zero magnetic field from the paramagnetic (ergodic) phase through the spin freezing temperature *T*_f_ into the nonergodic phase, where the cooling is stopped at a certain temperature *T*_1_ < *T*_f_ for a waiting (aging) time *t*_w_ ranging from minutes to hours and the spin system is left there to age isothermally. After *t*_w_, the cooling is resumed and eventually one or more consecutive stops are performed at a sequence of decreasing temperatures *T*_*i*_ < *T*_*i*–1_ < ··· < *T*_3_ < *T*_2_ < *T*_1_, where the spin system is aging isothermally at each stop temperature for arbitrary *t*_w_. After the last stop, the system is cooled continuously down to the lowest temperature of the experiment, where a tiny external magnetic field of the order μ_0_*H* = 0.1 mT is applied. The temperature sweep is then reversed and the zfc magnetization *M*_zfc_ is measured in a continuous heating run through *T*_f_ back to the ergodic phase. The spin system remembers its cooling history, which is manifested as a diminution (a dip) in the *M*_zfc_ at each stop temperature *T*_*i*_ relative to the “no-stop” reference zfc magnetization *M*_zfc_^0^. The frustrated spin system additionally remembers the duration of each stop, manifesting as an increased diminution of *M*_zfc_ for longer *t*_w_’s. After heating the spin system through *T*_f_ back to the ergodic phase, all of the stored information on the isothermal aging within the nonergodic phase is erased and the system becomes “young” (unaged) again, a phenomenon known as rejuvenation. The ME has already found application in a special kind of a memory element, a thermal memory cell,^34^ where a byte of digital information is stored into the magnetically frustrated nonergodic material by pure thermal manipulation using a specific temperature–time profile, in the absence of electric, magnetic or electromagnetic field.

**Figure 12:**
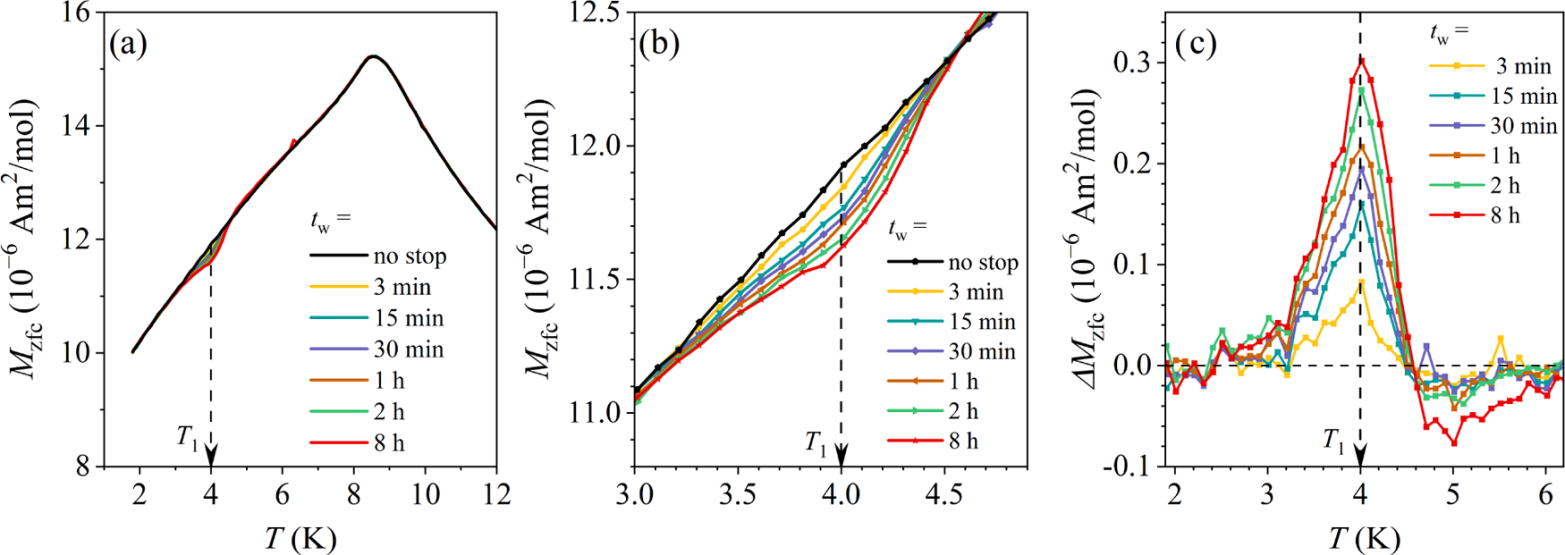
Memory effect in the *x* = 0.4 alloy. (a) *M*_zfc_ curves for different aging times *t*_w_ at the stop temperature *T*_1_ = 4 K. (b) Expanded portions of the *M*_zfc_ curves around the stop temperature. (c) Normalized difference between the no-stop magnetization and the aged magnetization ΔM = [*M*_zfc_^0^*– M*_zfc_ (*t*_w_)]/*M*_zfc_^0^, as a function of the aging time *t*_w_.

The ME in the *x* = 0.4 alloy was investigated by cooling the material in zero field to the stop temperature *T*_1_ = 4 K, where a series of aging times *t*_w_ between 3 min and 8 h was employed (each *t*_w_ in a separate experiment). The no-stop (*t*_w_ = 0) reference run was also carried out. After *t*_w_, the cooling was resumed to the lowest temperature of 1.8 K, where a magnetic field of 0.5 mT was applied and the zfc magnetization *M*_zfc_ was measured in a heating run. The resulting *M*_zfc_’s of all runs with different *t*_w_’s are shown superimposed in Figure 12a, whereas expanded portions of the same curves in the vicinity of the stop temperature 4 K are presented in Figure 12b. A diminution (a dip) in the aged *M*_zfc_ curves centered at the stop temperature is evident, where the dip increases in magnitude with the increasing *t*_w_. The normalized (dimensionless) difference between the no-stop (*t*_w_ = 0) reference magnetization *M*_zfc_^0^ and the aged magnetization *M*_zfc_ (*t*_w_), Δ*M* = [*M*_zfc_^0^*– M*_zfc_ (*t*_w_)]/*M*_zfc_^0^, as a function of *t*_w_ is presented in Figure 12c. A resonant bell-like shape of Δ*M* is evident, being peaked at the stop temperature of 4 K and having a width of about ±0.5 K on the temperature axis. Δ*M* increases systematically in magnitude with increasing *t*_w_.

**Figure 13:**
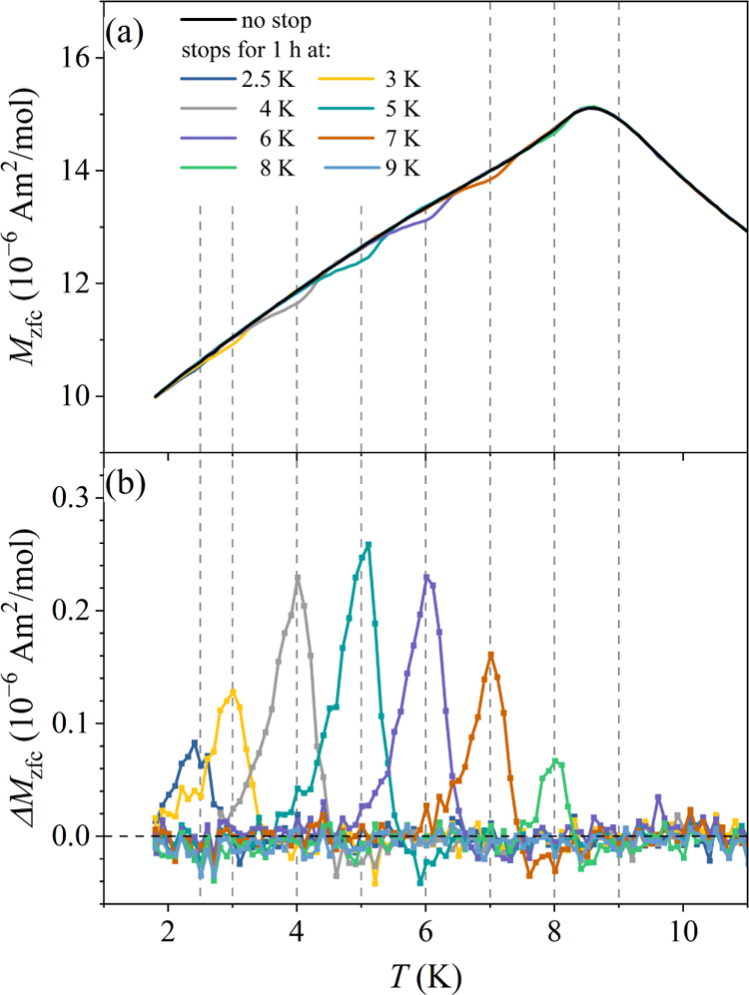
(a) *M*_zfc_’s of a set of single-stop experiments with the stop for *t*_w_ = 1 h at the temperatures *T*_*i*_ between 9 and 3 K in steps of Δ*T*_*i*_ = 1 K, and in addition at 2.5 K (the curves are superimposed on the same graph). (b) Normalized difference Δ*M* between the no-stop reference magnetization and the aged magnetization for each stop temperature.

The ME at different stop temperatures was investigated by a set of single-stop experiments with the stops at the temperatures *T*_*i*_ between 9 and 3 K in steps of Δ*T*_*i*_ = 1 K, and in addition at 2.5 K. The aging time *t*_w_ = 1 h was employed in each experiment. The *M*_zfc_ curves of all experiments are shown superimposed in Figure 13a, whereas the normalized difference Δ*M* between the no-stop reference magnetization and the aged magnetization for each stop temperature is presented in Figure 13b. As expected, there is no ME at 9 K, because at that temperature, the spins are still in the ergodic phase, whereas all aging stops within the nonergodic phase (at *T*_*i*_ ≤ *T*_f_ ≈ 8 K) have been memorized by the spin system.

## 4 Discussion

An important motivation to investigate the δ-Co_2.5_Zn_17.5–*x*_Mn_*x*_ pseudo-binary intermetallic system was searching for nontrivial magnetically ordered states that would result from chirality of the crystal structure of these alloys, such as spirally ordered helimagnetic and conical spin structures or skyrmion and chiral spin soliton lattices. Instead, by performing a thorough magnetic study of the Co_2.5_Zn_17.1_Mn_0.4_ substitutionally (chemically) disordered intermetallic compound, we have found an “excellent” spin glass with no relation to the structural chirality. This compound contains all of the necessary ingredients for the formation of the spin glass state: (a) randomness (a significant fraction of the Co moments and all Mn moments are randomly positioned in the crystal lattice) and (b) frustration (the competing FM and AFM interactions of the RKKY type prevent the spin system to achieve a configuration that would satisfy all of the bonds and minimize the free energy at the same time). The observed spin glass phase exhibits typical broken-ergodicity phenomena below the spin freezing temperature *T*_f_ ≈ 8 K: (i) there is a large difference between the zfc and fc susceptibilities in low magnetic fields, (ii) the frequency-dependent spin freezing temperature *T*_f_ (ν) depends logarithmically on the frequency ν of the applied ac magnetic field, (iii) the spin system shows remanence and hysteresis, (iv) the thermoremanent magnetization decays logarithmically slow in time, and (v) the memory effect was observed, where the nonergodic spin system memorizes its cooling history in zero magnetic field within the nonergodic phase. To this end, the investigated Co_2.5_Zn_17.1_Mn_0.4_ alloy is similar to other nonergodic, magnetically frustrated systems like site-disordered canonical spin glasses,^17,22−29,32,33^ site-ordered but geometrically frustrated quasicrystals and their periodic approximants,^30,35,36^ giant-unit-cell complex metallic alloys,^31^ high-entropy alloys,^37^ substitutionally disordered intermetallics^38^ and magnetic nanoparticles.^39,40^ All of these systems exhibit a more or less identical dependence of the thermoremanent magnetization and the memory effect on the aging temperature *T*_m_, aging time *t*_w_, and cooling field *H*_fc_, where these out-of-equilibrium phenomena are theoretically still incompletely understood.

The term “spin glass” refers to a broad class of magnetically frustrated systems with mixed FM and AFM interactions and randomly positioned spins. There exist subclasses of this general class, based on the sign of the average exchange coupling constant, either  (an asperomagnetic state),  (a speromagnetic state), or  (a “true” spin glass), which are difficult to distinguish on the basis of broken-ergodicity phenomena. The fact that the Curie–Weiss temperature (a measure of the average exchange coupling in the spin system) of the Co_2.5_Zn_17.1_Mn_0.4_ alloy is close to zero (θ = −4 K), is in favor of a true spin glass state.

The observation of the spin glass phase in the δ-Co_2.5_Zn_17.1_Mn_0.4_ alloy with the low Mn content that is in no relation to its structural chirality in principle does not preclude the existence of chirality-affected magnetic phases in the δ-Co_2.5_Zn_17.5–*x*_Mn_*x*_ alloys at higher Mn contents (within the existence range of the δ-phase, roughly at *x* ≈ 0–3.5). However, in view of the fact that the only chiral distribution of magnetic moments in the δ-Co_2.5_Zn_17.5–*x*_Mn_*x*_ structure are the Co atoms on the Co11 (6*c*) Wyckoff site, which form a double helix (see Figure S1b in the Supporting Information), while the Mn atoms are randomly located on the Zn/Mn mixed sites on the icosahedra, the increased Mn concentration increases the number of randomly positioned spins so that the formation of a chirality-induced magnetic structure in higher Mn-content alloys is even less likely. The most obvious candidate to search for a chirality-induced magnetic structure in this alloy system is the pure (*x* = 0) binary compound δ-Co_2.5_Zn_17.5_. The pure compound contains only one type of magnetic moments (the Co moments), but it also contains randomness because a significant fraction of the Co moments (those on the Zn8/Co8 mixed site) occupy that site randomly. To the best of the Authors’ knowledge, there is no report in the literature on the magnetism of the δ-Co_2.5_Zn_17.5_ pure compound at the time of writing, which remains the subject for future research.

## 5 Conclusions

We have synthesized the δ-Co_2.5_Zn_17.5–*x*_Mn_*x*_ (*x* = 0.4–3.5) pseudo-binary alloys of 10 different compositions by high-temperature solid-state synthetic route and determined their crystal structures, the Mn substitution pattern with regard to the δ-Co_2.5_Zn_17.5_ parent binary phase and estimated the existence range of the δ-phase. The investigated δ-Co_2.5_Zn_17.5–*x*_Mn_*x*_ alloys crystallize in two chiral enantiomorphic space groups *P*6_2_ and *P*6_4_ in the entire substitution range, where the basic atomic polyhedron of the structure is an icosahedron and the neighboring icosahedra share vertices to form an infinitely long double helix along the hexagonal axis (like in the δ-Co_2.5_Zn_17.5_ parent binary phase). The alloys are phase-pure up to the Mn content *x* ≈ 3.5, while at higher Mn contents, the cubic γ-brass type phase (*I*4̅_3_*m*) starts to form. The Mn atoms partially substitute Zn atoms at particular crystallographic sites located on the icosahedra.

The study of magnetism was performed on the Co_2.5_Zn_17.1_Mn_0.4_ alloy with the lowest Mn content. Contrary to the expectation that structural chirality may induce the formation of a nontrivial magnetic state, a standard spin glass state with no relation to the structural chirality was found. The magnetic sublattice contains all of the necessary ingredients (randomness and frustration) for the formation of a spin glass state. Typical out-of-equilibrium dynamic phenomena of a spin system with broken ergodicity were detected below the spin freezing temperature *T*_f_ ≈ 8 K.

## 6 Experimental Section

Single-crystal X-ray diffraction (SCXRD) measurements were carried out on single crystals picked up from each batch of loads. The crystals were mounted onto the goniometer head by adhering them to the glass fiber’s tip. A Bruker Photon II detector equipped with graphite-monochromatic Mo Kα radiation (λ = 0.71073 Å) was used to gather each batch of data at ambient temperature.

Powder X-ray diffraction (PXRD) data were collected at ambient temperature using Bruker D2 Phaser diffractometer with 600 W X-ray tube (Cu Kα_1_ radiation, λ = 1.54056 Å). The ingots were broken down and ground into a fine powder using an agate mortar and pestle. The data were collected for the 2θ range from 10 to 70° with a step size of 0.03°.

Energy-dispersive X-ray spectroscopy (EDS) analysis of the chemical composition was performed by the scanning electron microscope ThermoFisher Quanta 650 ESEM equipped with EDS Oxford Instruments AZtec Live, Ultim Max SDD 40 mm^2^ detection system.

Magnetic measurements were conducted on a Quantum Design MPMS3 magnetometer, equipped with a 7 T magnet and operating at temperatures between 1.8 and 400 K. Since the measurements of the thermoremanent magnetization and the memory effect were conducted in very low external magnetic fields of the order μ_0_*H* = 0.1 mT (equivalent to 1 G, in cgs units), we were using the Ultra-Low Field option to compensate for the residual magnetic field of the superconducting magnet, providing accuracy of the field setting to ±2 × 10^–3^ mT (equivalent to ±0.02 G in cgs). The low-field experiments were conducted using a copper AC/ULF coil of the MPMS3 magnetometer to ensure an accurate and repeatable magnetic field. A sample of dimensions about 5 × 2 × 2 mm^3^ was used, roughly resembling a spherical ellipsoid. Its long axis was set parallel to the magnetic field, to minimize the demagnetization effects.

## Supporting Information Available

Crystal structure and magnetic lattice of the δ-Co_2.5_Zn_17.5_ parent binary compound (Figure S1), and interatomic distances (<3.0 Å) for each atomic site of the Co_2.5_Zn_16.9_Mn_0.6_ (c2 composition) (Table S1) (PDF)
