# Supplementary material for: Double Helix of Icosahedra Structure and Spin Glass Magnetism of the δ-Co2.5Zn17.5–xMnx (x = 0.4–3.5) Pseudo-Binary Alloys
Source: Inorg Chem. 2024 May 20;63(22):10251–63. doi: 10.1021/acs.inorgchem.4c00686 (PMC11151197; doi:10.1021/acs.inorgchem.4c00686)
Supplement: Supplementary file 1 — ic4c00686_si_001.pdf [file ic4c00686_si_001.pdf]

Supporting Information

Double-helix of icosahedra structure and spin-glass magnetism of the δ-$Co_{2.5}Zn_{17.5-x}Mn_x$ (x = 0.4–3.5) pseudo-binary alloys

Amit Mondal,[1] Riju Dey,[1] Andreja Jelen,[2] Primož Koželj,[2,3] Sandip Kumar Kuila,[1] Rahul Pan,[1] Stanislav Vrtnik,[2] Jože Luzar,[2] Magdalena Wencka,[2,4] Julia Petrović,[2] Peter Mihor,[2] Zvonko Jagličić,[5] Anton Meden,[6] Partha Pratim Jana,[1] Janez Dolinšek[2,3]

[1] *Department of Chemistry, Indian Institute of Technology, Kharagpur-721302, India*

[2] *J. Stefan Institute, Jamova 39, SI-1000 Ljubljana, Slovenia*

[3] *University of Ljubljana, Faculty of Mathematics and Physics, Jadranska 19, SI-1000 Ljubljana, Slovenia*

[4] *Institute of Molecular Physics, Polish Academy of Sciences, Smoluchowskiego 17, PL-60-179 Poznań, Poland*

[5] *Institute of Mathematics, Physics and Mechanics & University of Ljubljana, Faculty of Civil and Geodetic Engineering, Jadranska 19, SI-1000 Ljubljana, Slovenia*

[6] *University of Ljubljana, Faculty of Chemistry and Chemical Technology, Večna pot 113, SI-1000 Ljubljana, Slovenia*

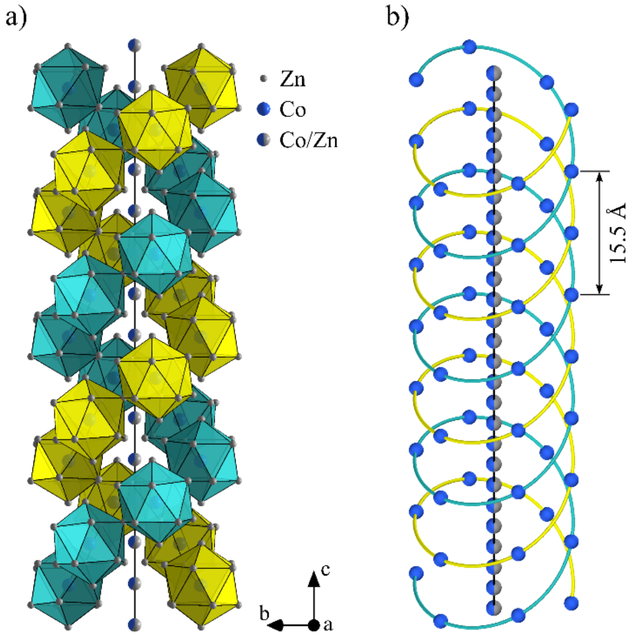

Fig. S1: (a) Crystal structure of the δ-$Co_{2.5}Zn_{17.5}$ binary intermetallic compound[1] (note that according to the PDF 04-017-0765 entry of the International Centre for Diffraction Data base, the δ-$Co_{2.5}Zn_{17.5}$ and δ-Co_2Zn_{15} chemical formulae refer to the same compound). **(b)** The magnetic lattice of the δ-$Co_{2.5}Zn_{17.5}$ binary compound, consisting of the Co magnetic ions only. The Co atoms from the icosahedra centers (the Co11 6*c* site) form a regular double-helix structure (with all sites fully occupied), while the Co atoms on the 6-axis (the mixed Zn8/Co8 3*a* site with the Zn/Co occupancies 0.58/0.42) are statistically randomly substituted by the nonmagnetic Zn, approximately half-half, introducing randomness into the magnetic lattice.

(1) Boström, M.; Lidin, S. Preparation and double-helix icosahedra structure of *δ*-Co_2Zn_{15}. *J. Solid State Chem.* **2002**, *166*, 53–57.

Table S1. The interatomic distances (< 3.0 Å) for each atomic site of the $Co_{2.5}Zn_{16.9}Mn_{0.6}$ (the c2 composition).

Zn1	Co11	2×	2.4907(80)
	Zn2	2×	2.5947(5)
	Zn6/Mn6	2×	2.6506(132)
	Zn4/Mn4	2×	2.6621(76)
	Zn5	2×	2.7147(132)
	Zn3	2×	2.7294(7)
Zn2	Co11	1×	2.4801(82)
	Co11	1×	2.5518(76)
	Zn1	1×	2.5947(5)
	Zn9/Mn9	1×	2.6496(9)
	Zn9/Mn9	1×	2.6538(143)
	Zn10	1×	2.6542(142)
	Zn10	1×	2.6703(12)
	Zn7	1×	2.6831(71)
	Zn4/Mn4	1×	2.7008(78)
	Zn6/Mn6	1×	2.8512(121)
	Zn5	1×	2.9439(124)
Zn3	Co11	1×	2.5696(78)
	Co11	1×	2.6406(73)
	Zn6/Mn6	1×	2.6639(20)
	Zn7	1×	2.6760(71)
	Zn4/Mn4	1×	2.6906(78)
	Zn5	1×	2.7054(13)
	Zn1	1×	2.7294(7)
	Zn3	2×	2.7678(142)
	Zn6/Mn6	1×	2.8586(121)

Zn3	Zn5	1×	2.9483(124)
Zn4/Mn4	Zn7	1×	2.5783(150)
	Co11	1×	2.6322(152)
	Zn1	1×	2.6621(76)
	Zn10	1×	2.6733(84)
	Zn5	1×	2.6767(89)
	Zn6/Mn6	1×	2.6851(87)
	Zn3	1×	2.6906(78)
	Zn2	1×	2.7008(78)
	Zn5	1×	2.7914(56)
	Zn9/Mn9	1×	2.8487(70)
	Zn5	1×	2.9366(54)
	Zn10	1×	2.9985(57)
Zn5	Co11	1×	2.5902(64)
	Zn10	1×	2.6727(11)
	Zn4/Mn4	1×	2.6767(89)
	Zn3	1×	2.7054(13)
	Zn5	1×	2.7222(11)
	Zn7	1×	2.7385(85)
	Zn7	1×	2.7466(85)
	Zn4/Mn4	1×	2.7914(56)
	Zn4/Mn4	1×	2.9366(53)
	Zn2	1×	2.9439(124)
	Zn3	1×	2.9483(124)
Zn6/Mn6	Zn1	1×	2.6506(132)
	Co11	1×	2.6515(58)
	Zn6/Mn6	1×	2.6547(9)
	Zn3	1×	2.6639(19)

	Zn9/Mn9	1×	2.6668(16)
Zn6/Mn6	Zn4/Mn4	1×	2.6851(87)
	Co11	1×	2.7459(56)
	Zn7	1×	2.7507(87)
	Zn7	1×	2.7537(87)
	Zn2	1×	2.8512(121)
	Zn3	1×	2.8586(121)
	Zn7	1×	2.8671(55)
Zn7	Co11	1×	2.5764(152)
	Zn4/Mn4	1×	2.5783(150)
	Zn3	1×	2.6760(72)
	Zn9/Mn9	1×	2.6827(76)
	Zn2	1×	2.6831(71)
	Zn5	1×	2.7385(85)
	Zn5	1×	2.7466(84)
	Zn6/Mn6	1×	2.7507(87)
	Zn6/Mn6	1×	2.7537(87)
	Zn6/Mn6	1×	2.8671(55)
Zn8/Co8	Zn10	2×	2.5666(71)
	Zn8/Co8	2×	2.5718(153)
	Zn9/Mn9	2×	2.6009(70)
	Zn10	2×	2.6568(79)
	Zn9/Mn9	2×	2.6918(79)
Zn9/Mn9	Zn8/Co8	1×	2.6009(70)
	Zn10	1×	2.6468(13)
	Zn2	1×	2.6496(9)
	Zn2	1×	2.6538(143)
	Co11	1×	2.6614(71)

	Zn6/Mn6	1×	2.6668(17)
Zn9/Co9	Zn7	1×	2.6827(76)
	Zn8/Co8	1×	2.6918(79)
	Zn10	1×	2.7881(151)
	Zn4/Mn4	1×	2.8487(70)
Zn10	Zn8/Co8	1×	2.5666(71)
	Co11	1×	2.6161(68)
	Zn9/Mn9	1×	2.6468(13)
	Zn2	1×	2.6542(142)
	Zn8/Co8	1×	2.6568(79)
	Zn2	1×	2.6703(12)
	Zn5	1×	2.6727(11)
	Zn4/Mn4	1×	2.6733(84)
	Zn9/Mn9	1×	2.7881(151)
	Zn4/Mn4	1×	2.9985(57)
Co11	Zn2	1×	2.4801(81)
	Zn1	1×	2.4907(80)
	Zn2	1×	2.5518(76)
	Zn3	1×	2.5696(78)
	Zn7	1×	2.5764(152)
	Zn5	1×	2.5902(64)
	Zn10	1×	2.6161(68)
	Zn4/Mn4	1×	2.6322(152)
	Zn3	1×	2.6406(73)
	Zn6/Mn6	1×	2.6515(58)
	Zn9/Mn9	1×	2.6614(71)
	Zn6/Mn6	1×	2.7459(57)
